# The Quantification of Drug Accumulation within Gram-Negative Bacteria

**DOI:** 10.1021/acsinfecdis.5c00921

**Published:** 2025-12-24

**Authors:** Amir George, Shivangi, Alexandra Bozan, Kendra Spencer, Austin J. Terlecky, Yong-Mo Ahn, Pamela R. Barnett, Barry N. Kreiswirth, Joel S. Freundlich

**Affiliations:** † Department of Pharmacology, Physiology, and Neuroscience, 5751 Rutgers University − New Jersey Medical School, Newark, New Jersey 07103, United States; ‡ 3139 Hackensack Meridian Health Center for Discovery & Innovation, Nutley, New Jersey 07110, United States; § Division of Infectious Disease, Department of Medicine and the Ruy V. Lourenco Center for the Study of Emerging and Re-Emerging Pathogens, Rutgers University − New Jersey Medical School, Newark, New Jersey 07103, United States

**Keywords:** gram-negative, bacteria, drug accumulation, drug metabolism, liquid chromatography, mass spectrometry

## Abstract

Intrabacterial drug accumulation, mediated by the bacterial permeability barrier, efflux, and intrabacterial drug metabolism, is of general significance to the interaction between small molecules and bacteria. For example, the ability of a small molecule to accumulate within a bacterium influences its ability to serve as a chemical probe of an intracellular protein target and/or its efficacy as an antibacterial drug discovery entity. A general method to quantitatively interrogate both intrabacterial drug accumulation and metabolism (IBDM) is presented for Gram-negative bacteria and exemplified with *Escherichia coli*, *Acinetobacter baumanni* *i*, *Klebsiella pneumoniae*, and *Pseudomonas aeruginosa* in both single-compound and high-throughput formats. The liquid chromatography–mass spectrometry-based platform does not depend on drug labeling, and its utility is highlighted through the exploration of the relationship between drug accumulation and drug minimum inhibitory concentration (MIC) for both wild-type and efflux-deficient strains of *E. coli* and *K. pneumoniae* clinical and laboratory strains of varying degrees of drug resistance. Furthermore, an investigation of drug synergy implicates the selective enhancement of the accumulation of one drug by its partner therapy. Finally, a high-throughput format is validated and deployed, which provides a readily adaptable approach to screening assays. We anticipate the further applications of this platform to both the translational and the fundamental studies of the interactions of small molecules with bacteria.

## Graphical abstract

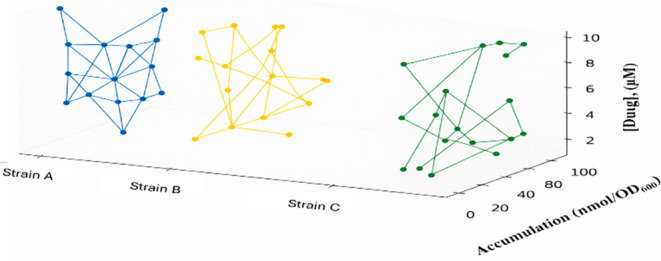

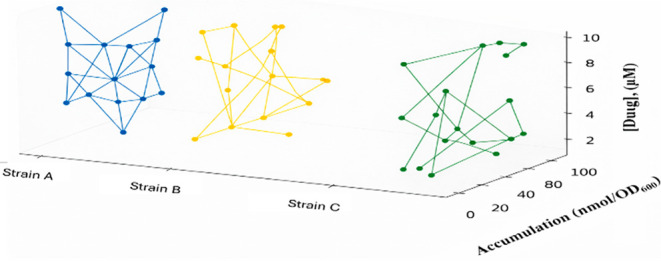

Antibacterial drug resistance represents a global health crisis.^1^ Left unchecked, annual global deaths are predicted to reach an estimated 10 million by 2050 and cost in the hundreds of trillions of US dollars.^2^ Gram-negative bacteria, in particular, represent a growing global health concern because of the emergence of multidrug-resistant (MDR) and pan-drug-resistant clinical isolates.^3^ Currently, the most serious infections are nosocomial and are typically caused by *Enterobacteriaceae* spp., *Pseudomonas aeruginosa*, and *Acinetobacter* spp.^4^ In addition, community associated multidrug resistant (MDR) infections are becoming increasingly prevalent and are most commonly associated with extended-spectrum β-lactamase-producing *Escherichia coli* and *Neisseria gonorrheae*.^4^ The United States Centers for Disease Control and Prevention has classified many of these pathogens as serious threats.^3^ Notable among the urgent risks are carbapenem-resistant *Enterobacteriaceae* (CRE) which cause over 9,000 infections annually in the United States and are estimated to have a 50% mortality rate.^3,5^ Many strains possess the *K. pneumoniae* plasmid encoding a carbapenem-resistant metallo-β-lactamase to confer significant resistance to β-lactams, including carbapenems, which are considered to be a treatment of last resort for MDR infections.^3,4^ In the United States alone, Gram-negative MDR pathogens have resulted in over 42,000 nosocomial infections per year, creating substantial economic strain on the healthcare system.^4^ However, in spite of the rising threat of MDR Gram-negative bacterial infections, only one new class of antibacterial (the triazaacenaphthylene gepotidacin) has been approved against these pathogens in the last 50 years.^6^

A major challenge in the fundamental studies of small molecule-bacteria interactions and in antibacterial chemotherapy is the consideration of the engagement of one or more intracellular protein targets by a small molecule.^7^ Bacteria have evolved highly effective defense mechanisms to counteract the presence of xenobiotics by limiting their accumulation within the cell itself (Figure 1). In Gram-negative bacteria, this accumulation hurdle is especially prominent^8^ and the cellular features of the resistance mechanism comprise: 1) a dual-membrane envelope, consisting of a lipopolysaccharide (LPS)-coated outer membrane, a thin layer of peptidoglycan, and a phospholipid bilayer inner membrane, acting as a formidable barrier to the penetration of small molecules;^9,10^ 2) an efficient multidrug efflux pump system from the intracellular environment;^11–13^ and 3) drug-metabolizing enzymes transforming the antibacterial into nontoxic metabolite/s.^14,15^ These features constitute a primary challenge to the discovery of novel antibacterials since reduced drug accumulation leads to diminished target engagement, affording attenuated whole-cell efficacy with or without an increased probability for the emergence of drug resistance. The issue of reduced drug accumulation may also be relevant to infection biology with regard to the consideration of persisters.^16^

**Figure 1:**
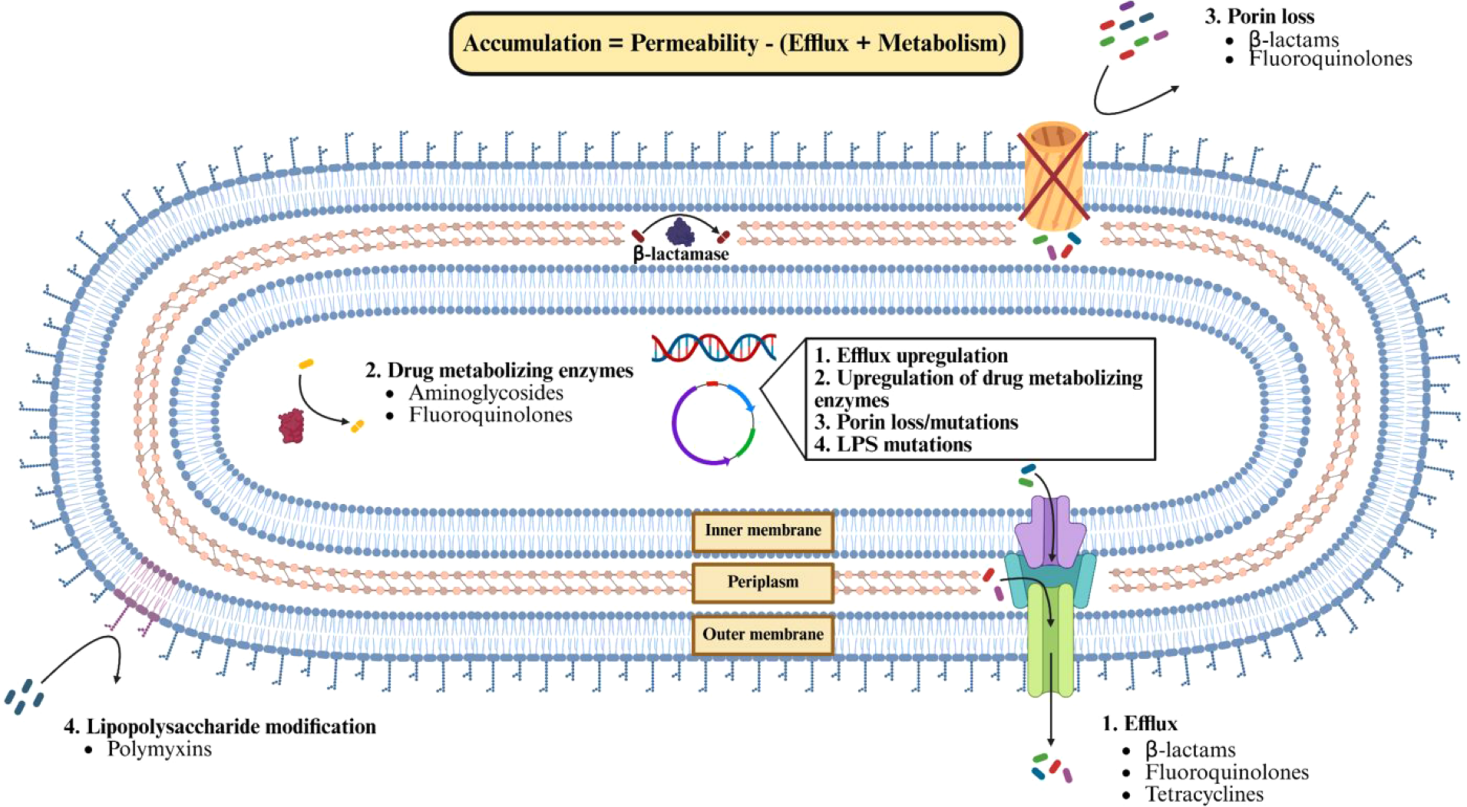
Factors affecting intrabacterial small molecule accumulation. An overview of factors that influence intrabacterial small molecule accumulation, including diffusion through porin channels, drug efflux and metabolism, and LPS modifications. Examples of drugs susceptible to these factors are listed. This figure was created with BioRender.

In this work, we begin with the presentation of a novel intrabacterial drug accumulation and metabolism (IBDM) protocol capable of directly interrogating the dynamics of both accumulation and metabolism within a range of Gram-negative bacteria in single-compound and high-throughput (htIBDM) formats. The experimental approach builds on previous studies from our laboratory with *Mycobacterium tuberculosis* ^17–20^ and Gram-positive bacteria^21^ while being complementary to published, mass spectrometry-based approaches discussed herein (with leading references from the Hergenrother^22^ and Tan^23^ laboratories with Gram-positive and Gram-negative bacteria as well as *Mycobacterium smegmatis*). We now report the study of the intrabacterial accumulation of parent drugs in a range of Gram-negative bacteria. Furthermore, we exemplify the intrabacterial metabolism of rifampicin in *E. coli*. Additionally, we detail drug accumulation measurements as to their underappreciated utility in the mechanistic study of drug synergy and their novel application to study the relationship between plasmid-mediated multidrug resistance and intrabacterial drug accumulation in a clinical isolate of *K. pneumoniae*.

## Results

### IBDM Assay Optimization with *E. coli*

We commenced with the adaptation of the IBDM assay from our previous work with *M. tuberculosis* ^17–20^ and *Staphylococcus aureus* ^21^ to pursue drug accumulation measurements in the wild-type *E. coli* strain MG1655. Four major experimental steps exist in the protocol: 1) bacterial growth, incubation with drug, and sample collection; 2) washing and quenching; 3) bacterial cell lysis and filtration; and 4) analysis of samples via liquid chromatography–mass spectrometry (LC/MS) featuring a single-quadrupole mass spectrometer (Figure 2). To optimize the protocol, bacteria were cultured in the presence of the antibacterial rifampicin (10 μM), and the effect of total culture volume (15, 25, and 125 mL) on intrabacterial accumulation was observed (Figure S1). No significant difference in rifampicin accumulation was noted as the volume was varied. Subsequently, bacteria were cultured in the presence of rifampicin or doxycycline (10 μM), and the effect of time point (10, 30, and 60 min) on accumulation was observed (Figure S2), where the time point was referenced to the addition of the drug to the bacterial culture. Rifampicin and doxycycline were utilized because they are low and high accumulating drugs in *E. coli*,^24^ respectively, and we hypothesized that their use in parameter optimization would result in a more sensitive and robust assay. Consistent with previous work, no significant difference in the accumulation of both compounds with respect to the three time points was found.^22,25^

**Figure 2:**
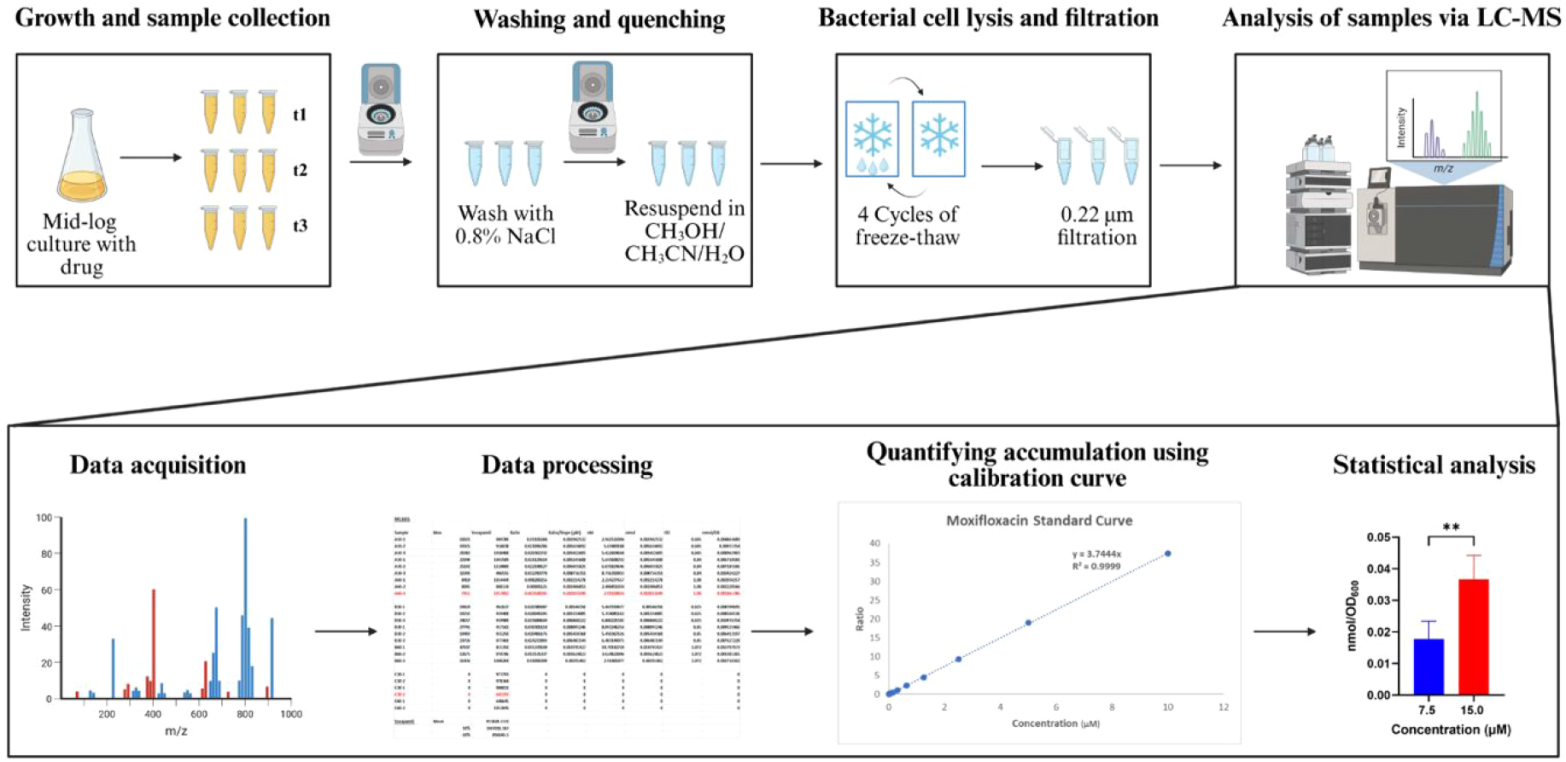
General IBDM assay protocol. Summary of the IBDM protocol, which allows for quantitative analysis of the accumulation of the drug and its identified metabolites within the bacteria under study. This figure was created with BioRender.

Subsequently, we sought to test the validity of using OD_600_ readings to normalize accumulation trends compared to colony-forming units (CFUs). We tested this by measuring the accumulation of doxycycline and rifampicin in wild type *E. coli*. No significant difference was observed between the two trends when comparing the different normalizations (Figure S3). We next observed changes in moxifloxacin, doxycycline, and rifampicin accumulation at the three different time points and two concentrations (10 and 20 μM). We observed no significant time-dependent changes in intrabacterial drug accumulation at 10, 30, and 60 min at the 10 μM concentrations for all drugs (Figure S4). However, a statistically significant difference in doxycycline accumulation between all three time points was observed at 20 μM. Therefore, all subsequent IBDM studies were pursued using the 10 and 60 min time points to account for potential time-dependent effects. All drugs exhibited significantly higher accumulation at the 20 μM dose compared to the 10 μM (Figure S5) suggesting dose-dependent accumulation for the drugs tested.

### Drug Accumulation of Doxycycline, Moxifloxacin, and Rifampicin in Isogenic Strains of *E. coli*

Since AcrAB-TolC is one of the major efflux pumps implicated in multidrug resistance in Gram-negative bacteria,^26^ we quantified the accumulation of this set of drugs in an efflux-deficient *ΔtolC* strain. We hypothesized intrabacterial drug accumulation would be increased in the *ΔtolC* strain for drugs that exhibit greater growth inhibition, i.e., a lower MIC, in this efflux-deficient strain as compared to the wild-type MG1655 strain. This was observed for both doxycycline and moxifloxacin (Figure 3) as their respective intrabacterial accumulations were significantly higher in the *ΔtolC* strain as compared to the MG1655 strain at the 60 min time point. Doxycycline and moxifloxacin are 16- and ≥ 16-fold more potent versus the *ΔtolC* strain as compared to the MG1655 strain, respectively (Table 1). No significant difference in rifampicin accumulation was observed between the wild-type and the *ΔtolC* strains at 10 μM (Figure 3). However, at 20 μM, there was a small (1.2x) but statistically significant difference in rifampicin accumulation favoring the *ΔtolC* strain over the wild-type strain, correlating with the modest change (2-fold) in MIC for rifampicin between the strains (Table 1). With respect to the two *E. coli* strains and three drugs, the changes in MIC and accumulation values exhibit an inverse correlation (Figure S6).

**Table 1:** MIC Values for Select Antibacterial Agents versus Wild-Type and Efflux-Deficient *E. Coli* ^a^

	*E. coli* strain MIC (μM)	
Drug	MG1655	*ΔtolC*
doxycycline	6.3	0.39
moxifloxacin	0.78	≤0.049
rifampicin	12	6.3

a Each MIC value represents the average value from a minimum of two independent experiments.

**Figure 3:**
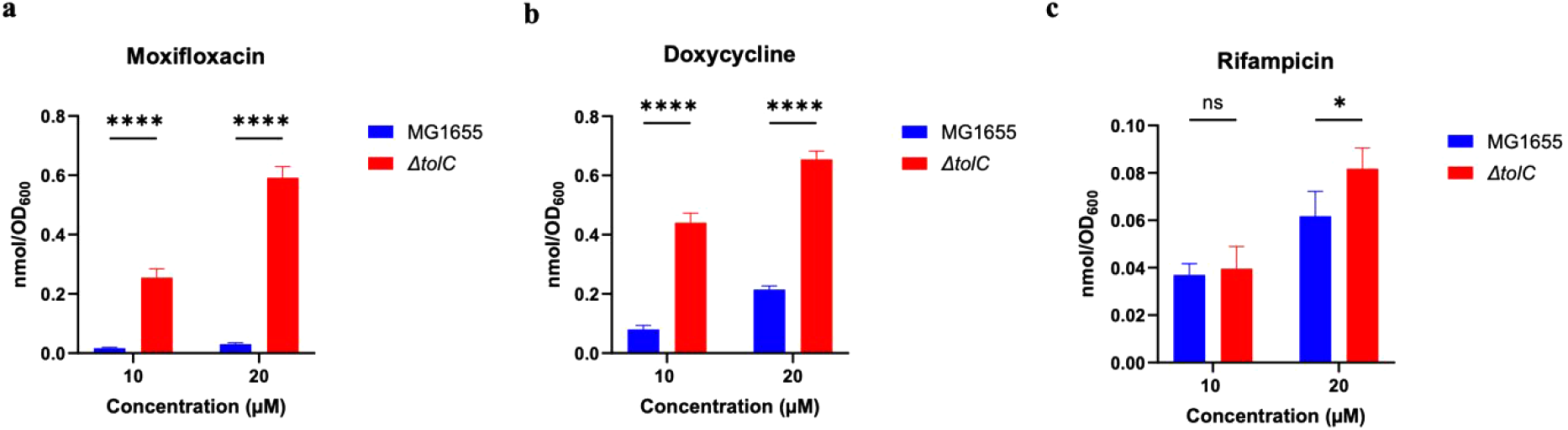
Accumulation of moxifloxacin, doxycycline, and rifampicin in *E. coli* MG1655 and Δ*tolC*strains. a. Doxycycline at 10 and 20 μM, b. Moxifloxacin at 10 and 20 μM, c. Rifampicin at 10 and 20 μM. All measurements were at *t* = 60 min. Data are shown as mean ± SD for a representative of two independent experiments, each conducted in quadruplicate. The amount of accumulated compound was quantified as the number of moles normalized by OD_600_. p-values were determined with two-way ANOVA with Šidák post hoc test. ns *p* > 0.05, * *p* < 0.05, ** *p* < 0.01, *** *p* < 0.001, **** *p* < 0.0001.

### An Intrabacterial Drug Metabolite within *E. coli* May Be Identified

Next, we sought to characterize any identifiable metabolites of rifampicin within *E. coli* MG1655. We focused on rifampicin due to the commercial availability of putative metabolites. MG1655 cells were treated with 200 μM rifampicin, and samples were collected after 10, 30, 60, and 90 min post-drug treatment. We identified one metabolite as rifampicin N-4′-oxide.^27^ It was characterized by the same mass spectrum and retention time as the commercial standard (Figure S7 and Table S1). The MIC value of rifampicin N-4′-oxide was quantified against *E. coli* MG1655 and it exhibited a significantly higher MIC value in comparison to rifampicin (Table S2), indicating the inactivation of the parent compound through drug metabolism.

### The IBDM Platform May Be Extended to Other Gram-Negative Bacteria

After leveraging the IBDM assay with *E. coli*, we sought to apply the platform to other Gram-negative bacteria of global health relevance. We selected representative strains of *Acinetobacter baumannii* (ATCC# 19606), *Pseudomonas aeruginosa* (ATCC# HER-1018), and *Klebsiella pneumoniae* (ATCC# BAA 2146) as they belong to a particularly difficult to treat group of bacteria associated with nosocomial infections known as ESKAPE bacteria. We chose rifampicin to assess against these bacteria due to its activity against the tested strains (Table S3). The *E. coli* IBDM protocol was utilized without alteration. Significantly higher accumulation of rifampicin at 10 μM compared to 1.0 μM was observed across all three bacteria, further validating the assay at detecting dose-dependent accumulation changes (Figure 4). At rifampicin concentrations of 1.0 and 10 μM for all three bacteria, we observed statistically significant greater accumulation of rifampicin in *A. baumannii* as compared to *K. pneumoniae* and *P. aeruginosa*. These results are consistent with the significantly greater potency of rifampicin versus *A. baumannii* in comparison to *P. aeruginosa* and *K. pneumoniae*.

**Figure 4:**
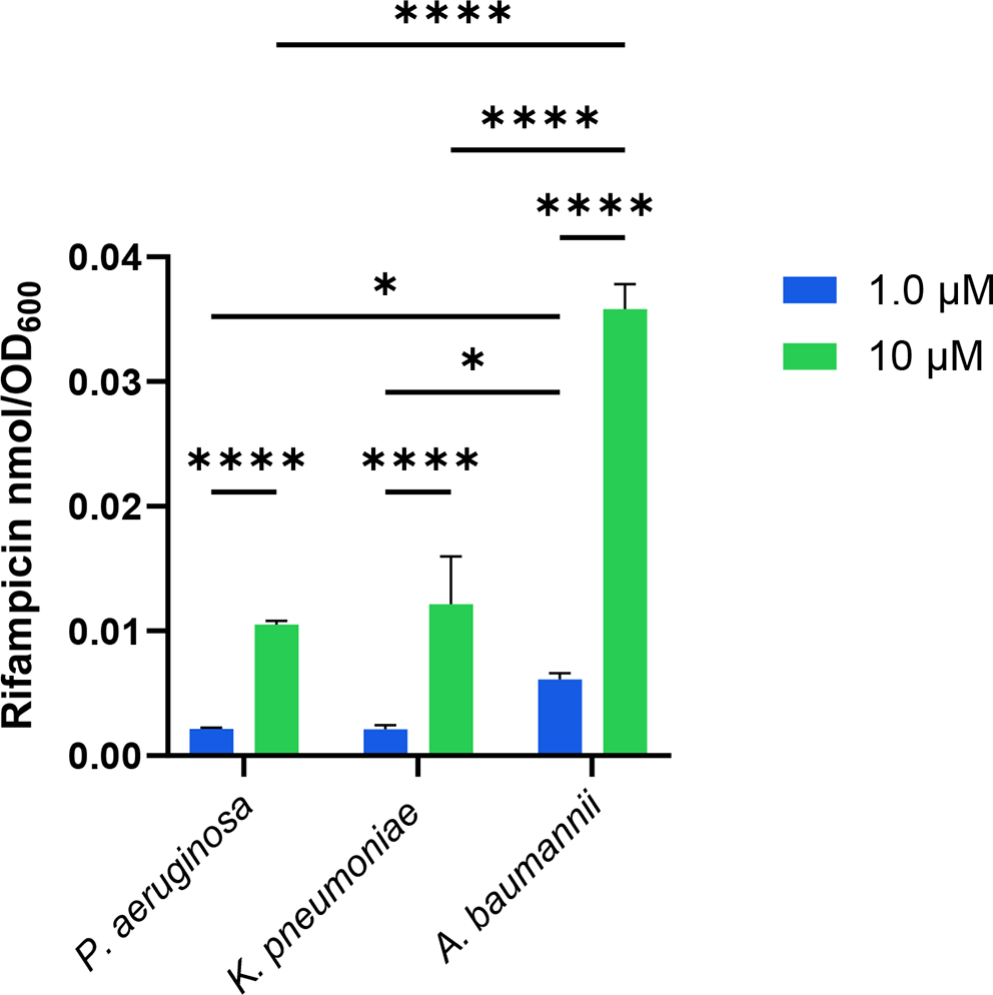
Rifampicin accumulation in *P. aeruginosa*, *K. pneumoniae*, and *A. baumannii*. Rifampicin (1.0 and 10 μM) accumulation in *A. baumannii* (ATCC# 19606), *P. aeruginosa* (ATCC# HER-1018), or *K. pneumoniae* (ATCC# BAA 2146) at *t* = 60 min. Data are shown as mean ± SD representative for a representative of two independent experiments each conducted in quadruplicate. The amount of accumulated compound as the number of moles was normalized by OD_600_. p-values were determined by two-way ANOVA with Šidák post hoc test. ns *p* > 0.05, * *p* < 0.05, ** *p* < 0.01, *** *p* < 0.001, **** *p* < 0.0001.

### Drug Accumulation Measurements May Be Applied to Examine Drug Combinations

Since drug combinations can be integral to the treatment of Gram-negative MDR infections, the IBDM assay was applied to study the intrinsic drug–drug interactions. A study from the Blainey laboratory^28^ showed that indacaterol – an approved drug for the treatment of chronic obstructive pulmonary disorder^29^ – exhibited synergy with novobiocin and erythromycin against the *E. coli* MG1655 strain. Indacaterol is inactive against this *E. coli* strain.^28^ Although novobiocin is active as a single agent versus Gram-positive bacteria, it is inactive against Gram negatives presumably due to its large size and hence low permeability across the Gram-negative membrane.^24,28,30^ Therefore, we hypothesized that indacaterol increases the permeability of *E. coli* through potential perturbation of the bacterial cell membrane. Given our previous profiling of rifampicin as a relatively low accumulating drug within *E. coli* MG1655, we chose to examine its interaction with indacaterol. A checkerboard assay was performed with rifampicin and indacaterol. The fractional inhibitory concentration index (FICI)^31^ was found to be ≤ 0.19 (Figure S8), classifying the combination as synergistic (i.e., FICI ≤ 0.5). After confirming the synergistic interaction between rifampicin and indacaterol, we quantified the accumulation in the *E. coli* MG1655 strain of rifampicin alone and in the presence of indacaterol. In the presence of 50, 100, or 200 μM indacaterol, rifampicin accumulation was significantly higher than that with rifampicin alone (Figure 5). Furthermore, no statistically significant difference was noted between the accumulation of rifampicin alone or in the presence of 25 μM indacaterol, which is lower than indacaterol’s MIC in combination.

**Figure 5:**
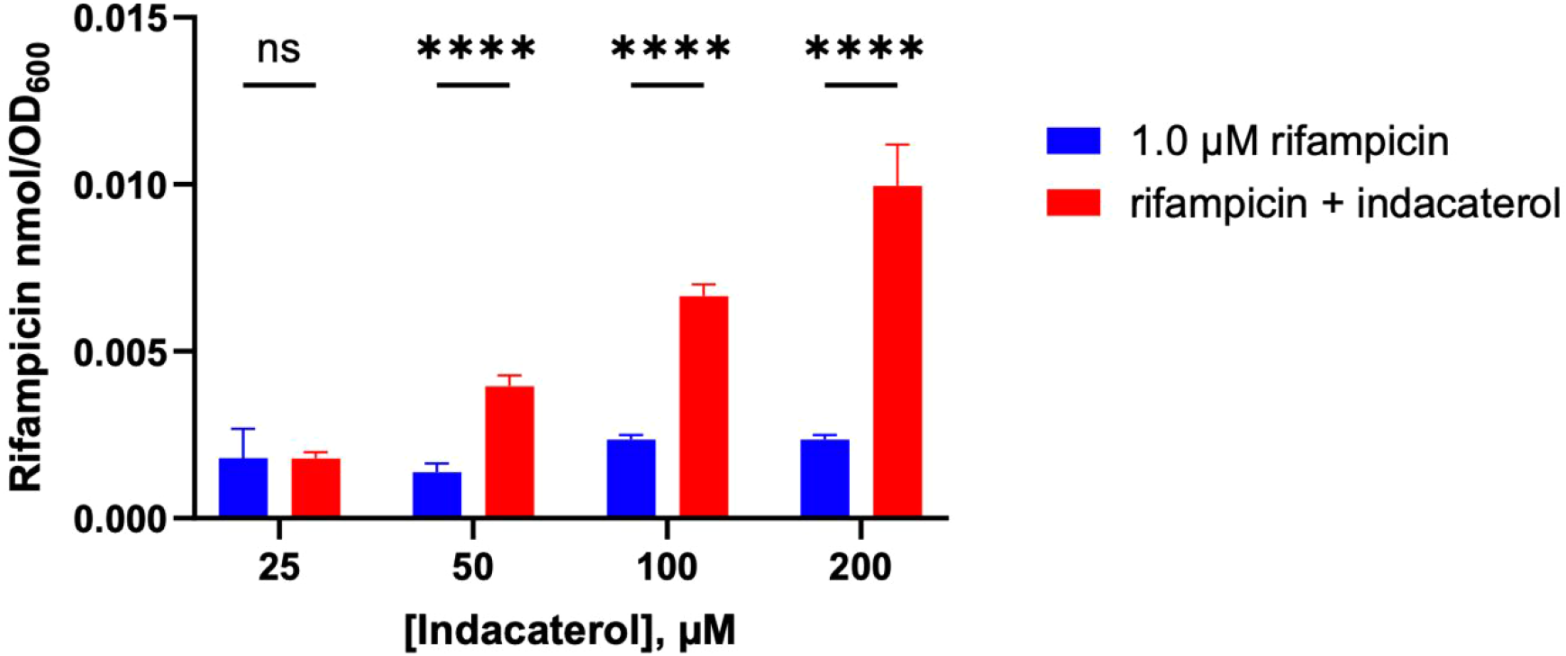
Rifampicin accumulation in *E. coli* MG1655 in the presence of indacaterol. Rifampicin (1.0 μM) in the presence of varying concentrations of indacaterol (25, 50, 100, or 200 μM) was incubated with *E. coli* MG1655 for 60 min prior to measurement. Data are shown as mean ± SD representative for a representative of two independent experiments each conducted in triplicate. The amount of accumulated compound as the number of moles was normalized by OD_600_. p-values were determined by two-way ANOVA with Šidák post hoc test for all assays. ns *p* > 0.05, * *p* < 0.05, ** *p* < 0.01, *** *p* < 0.001, **** *p* < 0.0001.

To further investigate this phenomenon, we performed flow cytometry studies using the cell-impermeable dye (TO-PRO-3). We observed a higher percentage of the cells taking up the dye at 200 μM indacaterol as compared to 25 μM indacaterol (Figure 6), and these results changed little when rifampicin and indacaterol were used in combination. Furthermore, we did not observe a significant difference in dye uptake between the 1.0 μM rifampicin and DMSO control treatments (Figure S9), suggesting that rifampicin is not affecting cell permeability alone and that the permeabilization observed in the 1:200 rifampicin:indacaterol condition is likely mediated through indacaterol. Altogether, these results are consistent with the hypothesis that indacaterol is synergizing with rifampicin through membrane permeabilization to increase the rifampicin accumulation inside the cells.

**Figure 6:**
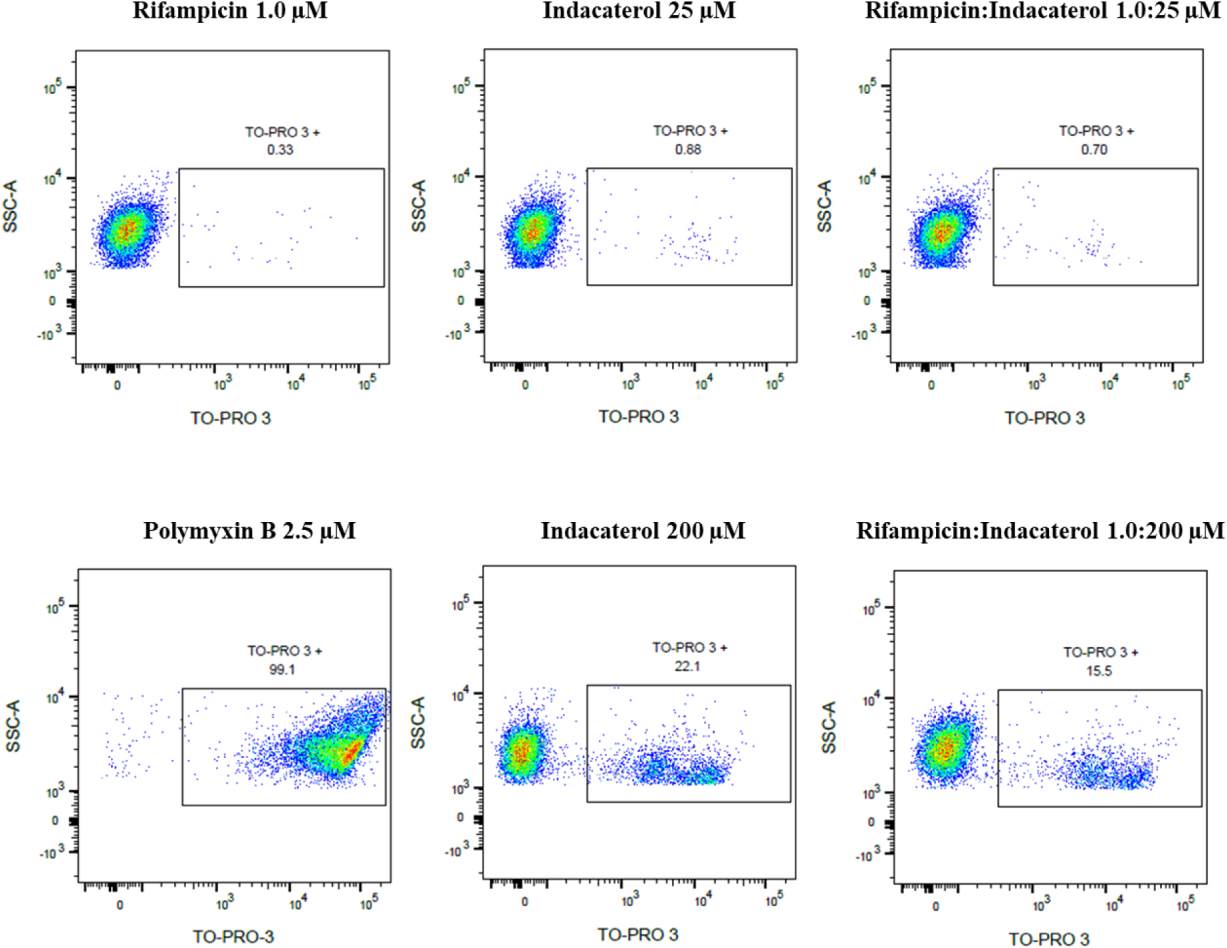
Effect of indacaterol on *E. coli* membrane permeability to TO-PRO-3 dye measured by flow cytometry. The percentage of cells that took up the dye is shown under each condition. The *y*-axis represents the side scatter area, and the *x*-axis represents the fluorescence of TO-PRO-3. Polymyxin B was used as the positive control. The results are representative of three independent experiments.

### A High-Throughput Platform (htIBDM) Is Developed

We next examined the possibility of extending the IBDM protocol to a high-throughput format (htIBDM). The protocol for htIBDM is similar to that for IBDM with a few alterations in the washing and lysis steps (Figure 7). To validate the htIBDM platform with a 96-well format, we calculated the Z′ value^32^ to be 0.51 with the *E. coli* MG1655 strain and 10 μM doxycycline (and DMSO as the negative control). We subsequently performed an htIBDM assay with doxycycline, moxifloxacin, ciprofloxacin, rifampicin, and novobiocin at concentrations of 20 and 40 μM with the *E. coli* MG1655 and *ΔtolC* strains at the 60 min time point (Figure 8). Accumulation trends were consistent when compared with results from single-compound IBDM for those same drugs (Figure S10), and these trends are also consistent with those reported by Richter and colleagues.^22^ Doxycycline was the highest accumulator in both assay formats, and rifampicin was the lowest accumulator. Ciprofloxacin accumulation was significantly higher than moxifloxacin accumulation in both wild-type and efflux-deficient *E. coli* despite both drugs belonging to the fluoroquinolone family, suggesting even seemingly minor differences in chemical structures can significantly impact accumulation. Novobiocin accumulation was lower in the htIBDM assay than in the IBDM assay, potentially due to an extra aqueous NaCl wash deemed necessary due to the comparatively higher predicted hydrophobicity of novobiocin (predicted h_logP^33^ of 3.4, Table S4) leading to its proposed nonspecific adherence to the membrane. Increasing the number of aqueous NaCl washes to two mitigated this problem (Figure S11).

**Figure 7:**
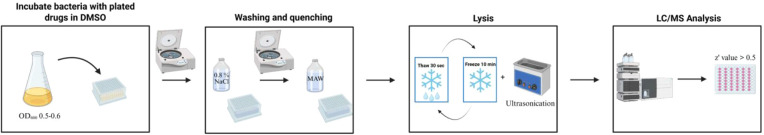
General high-throughput IBDM (htIBDM) protocol. Summary of the htIBDM protocol, which allows for quantitative analysis of the accumulation of drugs, as well as identified metabolites, in a 96-well plate. This figure was created with BioRender.

**Figure 8:**
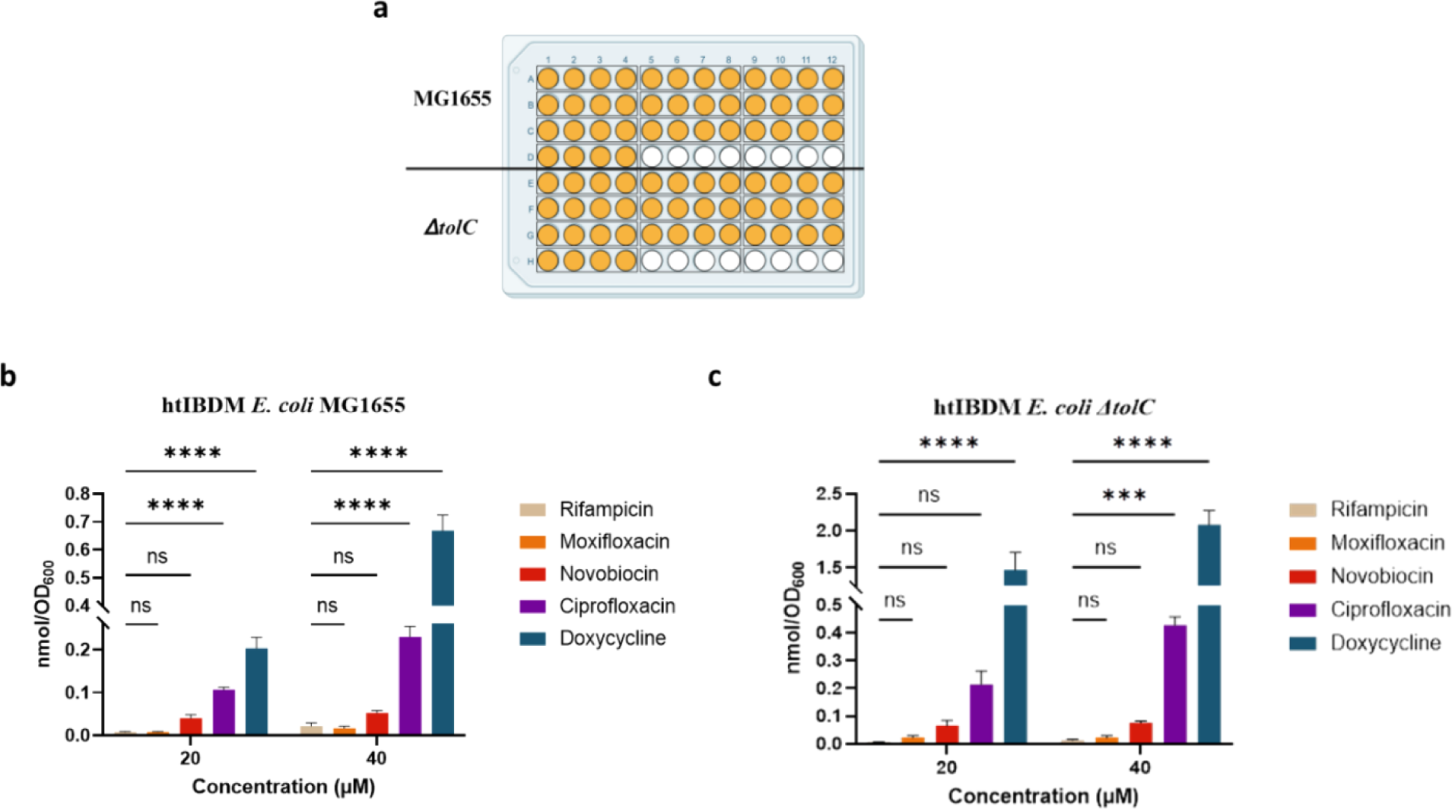
An exemplary htIBDM assay of select antibacterials with the *E. coli* MG1655 and Δ*tolC* strains. a. Plate layout for htIBDM to measure the intrabacterial accumulation of rifampicin, moxifloxacin, novobiocin, ciprofloxacin, and doxycycline at 20 and 40 μM (yellow-orange) and DMSO-treated negative controls (white) in quadruplicate with two *E. coli* strains at *t* = 60 min. Intrabacterial accumulation of drugs in b. wild-type MG1655 and c. efflux-deficient ΔtolC *E. coli* strains.Data are shown as mean ± SD representative of two independent experiments each conducted in quadruplicate. The amount of accumulated compound as the number of moles was normalized by cell number as approximated by OD_600_. p-values were determined by two-way ANOVA with Tukey post hoc test for all assays. ns *p* > 0.05, * *p* < 0.05, ** *p* < 0.01, *** *p* < 0.001, **** *p* < 0.0001.

The htIBDM assay was subsequently deployed to studies with *A. baumannii* (ATCC #19606), *P. aeruginosa* (ATCC #HER-1018), and *K. pneumoniae* (ATCC #BAA 2146). We examined the intrabacterial accumulation of doxycycline, moxifloxacin, ciprofloxacin, and rifampicin at a 20 μM concentration (Figure 9). Across all three strains and consistent with studies conducted in *E. coli* (Figure S10), doxycycline was the highest accumulator, rifampicin was the lowest accumulator, and ciprofloxacin accumulated higher than moxifloxacin. The accumulation of both doxycycline and ciprofloxacin was significantly lower in the *K. pneumoniae* strain used. This particular strain is a clinical isolate that exhibits multidrug resistance mediated through a combination of genes on its own chromosome and 23 plasmid-borne genes encoding drug resistance proteins.^34,35^ These observations piqued our interest in further exploring the effect of plasmid-mediated resistance on accumulation.

**Figure 9:**
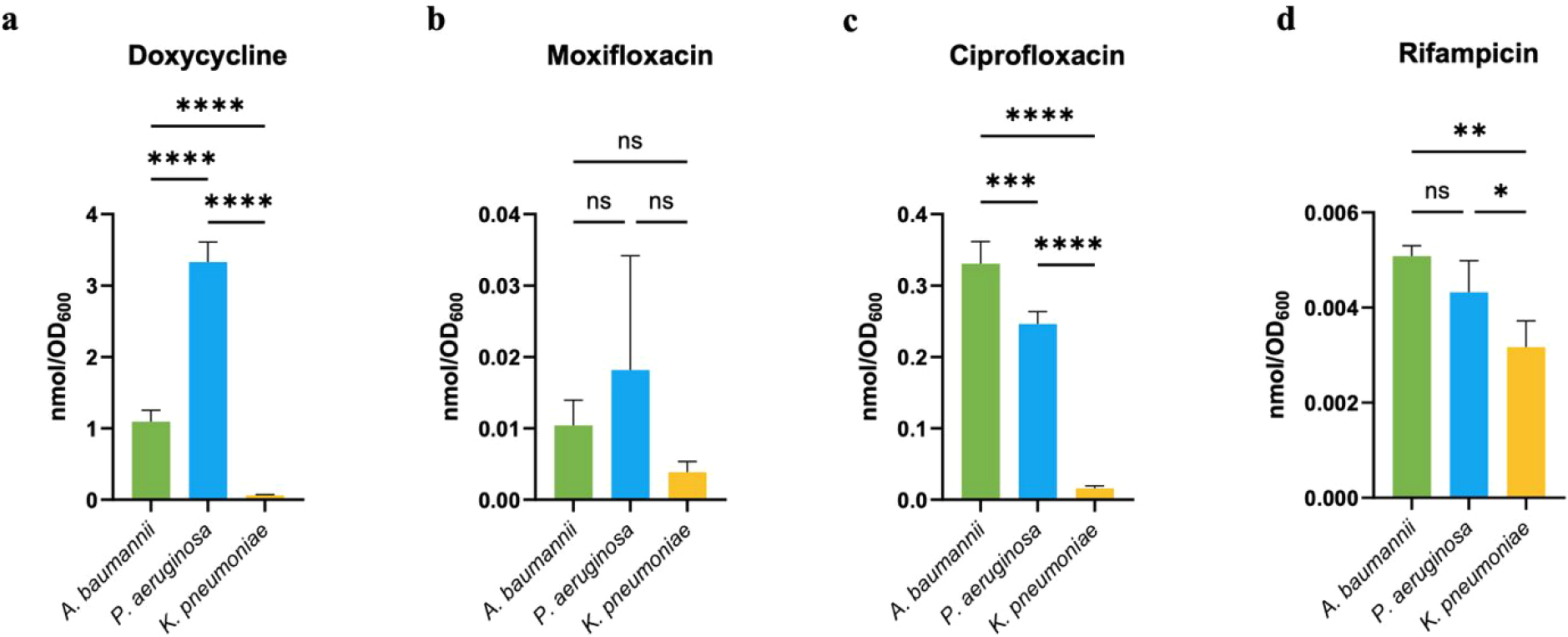
htIBDM profiling of antibacterial drugs with select ESKAPE strains. Intrabacterial accumulation at *t* = 60 min of a. doxycycline, b. moxifloxacin, c. ciprofloxacin, and d. rifampicin (20 μM) in *A. baumannii* (ATCC# 19606), *P. aeruginosa* (ATCC# HER-1018), or *K. pneumoniae* (ATCC# BAA 2146). Data are shown as mean ± SD representative of two independent experiments each conducted in quadruplicate. The amount of accumulated compound as the number of moles was normalized by cell number as approximated by OD_600_. p-values were determined by one-way ANOVA with Tukey post hoc test for all assays. ns *p* > 0.05, * *p* < 0.05, ** *p* < 0.01, *** *p* < 0.001, **** *p* < 0.0001.

### The htIBDM Assay Characterizes Plasmid-Mediated Multidrug Resistance in a Clinical Isolate of *K. pneumoniae*

To explore the effects of plasmid-mediated resistance in a representative MDR clinical isolate of *K. pneumoniae*, we set out to utilize the htIBDM platform to study drug accumulation in an MDR *K. pneumoniae* strain (70163), its plasmid-cured derivative strain (74189),^36^ and a *qnrB1* knockout strain (75762) derived from 70163 where *qnrB1* is a gene involved in fluoroquinolone resistance with poorly understood function.^37^ Using our previously disclosed CRISPR-Cas9 system technology,^36^ curing of the Inc.F hybrid plasmid (pKPN-K7)^38^ facilitated removal of all resistance genes harbored on it, including *aac­(6’)-Ib*-cr^39^ and *qnrB1* (Table S5). 70163 still contains known chromosomal mutations (GyrA-83I and ParC-80I) conferring fluoroquinolone resistance that persist within the plasmid-cured strain 74189. The observation that ciprofloxacin’s intrabacterial accumulation was higher than that for moxifloxacin in previous experiments with the *K. pneumoniae* ATCC# BAA 2146 strain (Figure 9) and the structural diversity within the fluoroquinolone family of drugs led us to hypothesize that accumulation occupies an important role in resistance to different fluoroquinolones.

Seven fluoroquinolones of clinical relevance were assessed for their MIC versus each of these three strains with doxycycline, rifampicin, and imipenem as control drugs (Table 2). The 70163 strain was not susceptible to any of the seven fluoroquinolones tested. In contrast, the plasmid-cured 74189 strain demonstrated a dramatic (≥8-fold) reduction in the fluoroquinolone MIC values as a likely consequence of losing both the *qnrB1* and *aac­(6’)-lb-*cr genes harbored on the plasmid. Furthermore, for all of the fluoroquinolones except norfloxacin, significant differences, of at least 4-fold, in the MICs were observed between the plasmid-containing strain 70163 and its derived *qnrB1* knockout strain.

**Table 2:** MIC Values for Select Antibacterial Agents versus an MDR *K. pneumoniae* Clinical Isolate (70163), Its *qnrB1* Knockout Strain (75762), and Its Plasmid-Cured Strain (74189)^a^

	*K. pneumoniae* strain MIC (μM)		
Drug	70163	75762	74189
ciprofloxacin	≥200	50	6.3
norfloxacin	>200	>200	25
gemifloxacin	50	12.5	6.3
ofloxacin	100	12.5	12.5
levofloxacin	50	12.5	6.3
moxifloxacin	50	12.5	6.3
delafloxacin	100	3.1	1.6
doxycycline	50	50	3.1
rifampicin	25	25	12.5
imipenem	1.6	0.78	1.6

a Each MIC value represents the average value from a minimum of two independent experiments in mm.

The fluoroquinolones tested may be generally stratified into two groups with regard to the three strains: high accumulators and low accumulators (Figure 10). The accumulations of delafloxacin were qualitatively intermediate between these two groupings. The high accumulators were ciprofloxacin, norfloxacin, and gemifloxacin and the low accumulators were levofloxacin, ofloxacin, and moxifloxacin. Of the three high accumulators, ciprofloxacin and norfloxacin showed statistically significant decreased accumulation in the MDR plasmid-containing strain (70163) as compared to the plasmid-cured strain (74189), suggesting that decreased intrabacterial accumulation could be a significant contributing factor to the strain’s resistance to those two agents. For all of the fluoroquinolones assessed for accumulation in the 75762 versus 70163 strains, we note that the deletion of *qnrB1* fails to result in statistically significant increased drug accumulation. This observation does not support a role for this gene in drug resistance through direct modulation of fluoroquinolone accumulation.

**Figure 10:**
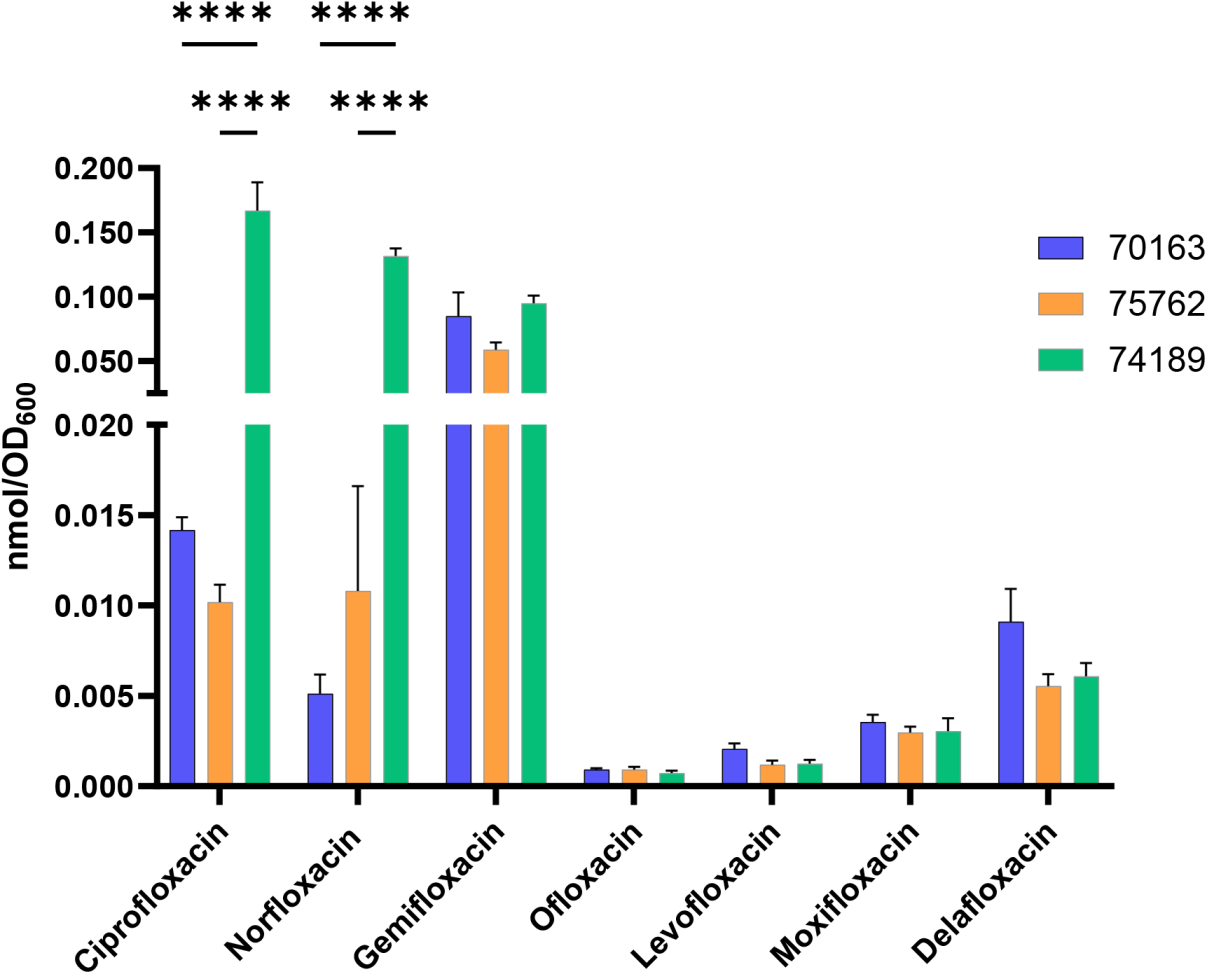
htIBDM assay of select antibacterials with a *K. pneumoniae* clinical isolate and its plasmid-cured and *qnrB1* knockout derivative strains. Intrabacterial accumulation of ciprofloxacin, norfloxacin, gemifloxacin, ofloxacin, levofloxacin, moxifloxacin, and delafloxacin (20 μM) in an MDR strain (70163), its plasmid-cured strain (74189), and its *qnrB1* knockout strain (75762) at *t* = 60 min. Data are shown as mean ± SD representative of two independent experiments each conducted in quadruplicate. The amount of accumulated compound as the number of moles was normalized by cell number as approximated by OD_600_. p-values were determined by two-way ANOVA with Šidák post hoc test for all assays. ns *p* > 0.05, * *p* < 0.05, ** *p* < 0.01, *** *p* < 0.001, **** *p* < 0.0001. Only statistically significant comparisons among accumulations for a given drug are annotated.

Cognizant of reported cheminformatic analyses and machine learning modeling of intrabacterial drug accumulation screens with variations in the number of compounds explored and their inherent diversity,^22,25,40–45^ we sought to explore the relationship between the calculated physicochemical properties of six fluoroquinolones studied and their accumulation values in the *K. pneumoniae* strain 70163 (Figure S12). Ofloxacin was omitted from the analysis since it is the racemate of levofloxacin. Physicochemical properties were selected based on previous reports correlating increased hydrophobicity and molecular weight with decreased intrabacterial accumulation in Gram-negative bacteria^40^ and the eNTRy rules developed by Richter et al. for *E. coli*.^41^ With the fluoroquinolones tested, a significant correlation between molecular weight and accumulation was not observed (R^2^ = 0.0047). However, an inverse correlation between h_logP and accumulation (R^2^ = 0.64) was noted. With respect to accumulation, the number of rotatable bonds demonstrated a significant correlation (R^2^ = 0.80) while globularity did not (R^2^ = 0.34). Finally, we note that gemifloxacin was the only fluoroquinolone assessed that contained an aliphatic, sterically unhindered primary amine and it had the highest intrabacterial accumulation in the 70163 strain.

## Discussion

Various methods have been previously reported to measure intrabacterial drug accumulation.^46^ Examples include radiometric detection,^47^ fluorescence quantification,^48,49^ and LC/MS-based assay.^22,23^ The LC/MS-based approaches typically have employed a triple-quadruple mass spectrometer which is more expensive and less prevalent in laboratories than the single-quadrupole instrumentation featured in our platform. Drug metabolism has not been generally quantified within a bacterium beyond a set of examples from our laboratory and others.^18,19,21,50–52^ We assert that an LC/MS-based assay is best suited to provide a direct, label-free quantification of intrabacterial drug accumulation and metabolism while radiometric detection necessitates the synthesis of radiolabeled drug which can be expensive and time-consuming. Furthermore, parent drug and drug metabolites may have the same response profile and may not easily be distinguished. Fluorescence-based measurements are limited by requiring the drug molecule being sufficiently fluorescent or necessitate the addition of a fluorescent moiety that may perturb the parent molecule’s biological activity and/or permeability.

Inspired by the well-established metabolomics platforms originating with Botstein,^53^ Rabinowitz,^53,54^ Rhee,^55^ and a subsequent report from the Dartois laboratory quantifying drug accumulation in *M. tuberculosis*,^56^ we published in 2019 the results of a long-standing effort to design the IBDM platform for *M. tuberculosis*. Our platform represented an amalgamation of the liquid culture approach of Dartois and the sample processing workflows of Botstein, Rabinowitz, and Rhee. Through studies focused on the mechanism of action of two series of nitro-containing heterocyclic antituberculars,^18,19^ we confirmed that our IBDM platform is a useful tool to monitor intrabacterial drug accumulation and metabolism in *M. tuberculosis*. Extensions of the platform were then achieved with *S. aureus* ^21^ and uninfected and *M. tuberculosis*-infected J774.1 macrophage-like cells.^17,20^ For Gram-negative bacteria, previous reports from other groups have focused on the development of a drug accumulation methodology and we highlight examples from Hergenrother (e.g., *E. coli* ^22^ and *P. aeruginosa* ^57^), Kern (e.g., *E. coli* ^58^), Stover (e.g., *P. aeruginosa* ^59^), Tan (e.g., *E. coli* ^23^ and *P. aeruginosa* ^60^), Zgurskaya (e.g., *E. coli*,^45^ *P. aeruginosa*,^44,45^ and *A. baumannii* ^45^), Erwin (e.g., *E. coli* ^25^) and Ritz (e.g., *E. coli* ^42^) that are label-free in that they do not require a reactive tag on the molecule to quantify its accumulation. In contrast are platforms that require a tag on the analyte drug such as a chloroalkane, a sulfide-linked d-cystine, or azide.^48,49,61^ However, we note the advantage of these approaches to examine localized accumulation (e.g., the cytosol or periplasm). Just as has been shown for the azide-tagged approach,^48^ our IBDM approach can be performed in high-throughput screening format. Validated herein with an acceptable Z’ factor, the htIBDM assay allows the rapid examination of an experimental matrix comprised of a range of both strains and compounds. We note that this 96-well assay format may be best utilized as a screen with the follow-up of “hits” or interesting results in the single-compound IBDM format. This guidance is supported by noting that the rank-ordering of drugs is roughly captured when comparing the htIBDM assay results (Figure 8) with those of the single-compound IBDM format (Figure 3). With this drug accumulation screening goal in mind, studies have been reported by Widya and colleagues.^62^ This approach, leveraging solid-phase extraction/mass spectrometry, provides excellent throughput while requiring specialized instrumentation that may not be present in the typical academic laboratory. The Zgurskaya laboratory has also applied this technology,^45^ while Erwin adapted^25^ an approach similar to that of Hergenrother into a 96-well format with a triple-quadruple mass spectrometer. The approach of Ritz^42^ is more similar to what we disclose herein but leveraged quadrupole time-of-flight mass spectrometry. Our htIBDM assay only requires a single-quad LC/MS instrument. It should be noted that the methodology used by Hergenrother differs from our approach in the utilization of 1) minimal media for the cell culture step in the presence of drug, 2) silicone oil in the cell pelleting step, and 3) CFUs to normalize quantified accumulation. Finally, although we have chosen to exemplify the htIBDM assay with a small number of compounds and two or more bacterial strains in a 96-well plate, one can facilely envision screening a larger number of small molecules for accumulation in a single bacterial strain.

A primary exemplification of our technology reported herein involves an investigation of the correlation of measurements of drug accumulation in Gram-negative bacteria with the quantifications of *in vitro* drug efficacy and resistance. We demonstrated with *E. coli* and a small set of antibacterials that changes in drug accumulation inversely correlate with the drug MIC when comparing bacterial strains (e.g., wild type MG1655 versus efflux deficient *ΔtolC*). Furthermore, comparison of the *in vitro* efficacy and accumulation profiles for a subset of approved fluoroquinolone drugs with the *K. pneumoniae* MDR clinical strain 70163 and its plasmid-cured derivative strain 74189 demonstrated that removal of the plasmid with known resistance genes can lead to the expected increases in drug susceptibility and drug accumulation. This observation was best demonstrated with ciprofloxacin and norfloxacin; although we note that the trend was not observable for four of the seven approved fluoroquinolones tested. We suspect that their differing accumulation behavior may be explained with further contemplation of the effect of their varying chemical substitutions of the fluoroquinolone scaffold. This knowledge, coupled with an understanding of their respective intracellular target engagement/s, should be useful in developing a better understanding of their drug resistance profiles. These studies add to a set of examples in the literature led by the Zgurskaya laboratory where 1) differences in compound growth inhibition as quantified by MIC have been compared to direct measurements of drug accumulation with a single bacterial strain^45,60,63–66^ and 2) quantitative changes in drug accumulation were correlated with mutations that affect either the outer membrane or efflux,^44,45,63,67^ porins,^44,45^ or transporters.^68^ Interestingly, we demonstrated that the CRISPRi knockdown of *qnrB1* affects the MIC values of the fluoroquinolone class without significantly perturbing accumulation. This outcome is consistent with the lack of data linking this gene to fluoroquinolone accumulation.^37^ These results with *K. pneumoniae* underscore the potential of our approach to illuminate the role of Gram-negative bacterial genes in drug resistance as viewed through the lens of drug accumulation. In particular, we see the promise of combining high-throughput genetic approaches such as CRISPRi^69^ with our htIBDM platform.

Another application of our technology is in the realm of drug combination studies. We have probed the interaction between rifampicin and indacaterol, inspired by the elegant study of Blainey leveraging a high-throughput nanoliter scale screen.^28^ In a dose-dependent fashion, we demonstrated the ability of indacaterol to augment the accumulation of rifampicin within *E. coli* and effectively increase the potency of rifampicin. While molecules such as colistin^22^ and pentamidine^70^ have been demonstrated to sensitize Gram-negative bacteria to typical Gram-positive selective agents, we are only aware of the study by Hergenrother that showed colistin to quantitatively augment the accumulation of novobiocin within the bacterium.^22^ We also note studies on compound accumulation in *E. coli* in the presence of efflux inhibitors.^23^ It is our expectation that further studies of the accumulation and metabolism dynamics of drug combination systems will be illuminated through the methods as outlined herein. It is intriguing to consider how traditional checkerboard studies used to infer interactions between two compounds, or their higher dimensional related approaches,^71^ could benefit from consideration of how combinations of drugs influence their individual accumulation within cells. Such knowledge gained from these studies should be critical to the rational development of combination therapies for diseases necessitating multidrug therapy.

Returning to the quantification of intrabacterial drug accumulation in single-compound studies, we speculate the field will be able to further stratify the results from high-throughput growth inhibition studies through the identification of compounds that do or do not achieve significant accumulation within the targeted cell. The generated screening data would augment currently available data sets for the construction and validation of machine learning models for drug accumulation and metabolism. Such computational approaches have been undertaken for *P. aeruginosa* ^43,44,72^ and *E. coli*.^22,42^ Compound accumulation guidances for *E. coli*, such as the installation of an unhindered, primary amine or the modulation of a compound’s number of rotatable bonds and globularity, have been suggested.^22^ However, we believe that, given other studies^42,57^ and our limited investigation of seven fluoroquinolones and *K. pneumoniae*, these approaches may be bacteria- and/or drug chemotype-specific. Specifically for the *K. pneumoniae* 70163 strain and six fluoroquinolones, we observed that intrabacterial accumulation increased with decreasing calculated logP (i.e., h_logP) and the increasing number of rotatable bonds. The compound molecular weight, globularity, and amphiphilic moment (i.e., vsurf_A) did not show significant correlations with drug accumulation. We expect that computational predictions in addition to the actual experimental determinations of drug accumulation should bolster antibacterial medicinal chemistry optimization to further dimensionalize and inform structure–activity relationship (SAR) studies. Early examples of this approach may be found in the literature,^60,64,65^ although they are far from commonplace.

Limitations to the methodology have already been discussed in detailed review of a somewhat similar protocol by Hergenrother,^73^ differing mainly in sample processing as discussed above. These limitations are related to the quantification of compound accumulation with an LC/MS based methodology, the potential for compound covalent attachment to intracellular proteins, the potential for nonspecifically bound, extracellular compound^23,25^ despite the current washing protocols (as discussed for both the single-compound and htIBDM assays), and an inability to provide information as to compound localization within the cell. Furthermore, with continued utilization of both the single-compound IBDM and 96-well plate htIBDM platforms, we expect to learn more about the influence of different assay parameters (e.g., number of washes) and drug properties (e.g, logP) on each platform’s ability to quantify drug accumulation.

## Conclusions

In conclusion, we reflect on the 2020 discussion of drug penetration in Gram-negative bacteria by Zgurskaya and Tan and their concluding remarks on remaining challenges.^74^ First, we assert the IBDM platform and the results discussed herein address the need for a high-throughput drug accumulation platform that is readily available to most laboratories. Second, our studies of drug resistance mutations in laboratory and clinical strains complement the authors’ publications in laying further groundwork for understanding how bacterial genes perturb drug accumulation and, hence, drug efficacy/resistance. These and other challenges as they pertain to small molecule-microbe interactions are open to being addressed through our platform in intrabacterial drug accumulation.

## Experimental Section

### Commercially Sourced Bacterial Strains and Reagents

*E. coli* K-12 MG1655 ^75^ and the isogenic efflux deficient strain Δ*tolC* ^76^ were obtained from the Brynildsen lab (Princeton University). *A. baumannii* (ATCC# 19606), *P. aeruginosa* (ATCC# HER-1018), or *K. pneumoniae* (ATCC# BAA 2146) were sourced from the ATCC. *K. pneumoniae* strain 70163 was accessed from the Hackensack Meridian Health Center for Discovery & Innovation clinical collection. Small molecules were sourced as follows: Chem-Impex – doxycycline, moxifloxacin, ciprofloxacin, and ofloxacin; MedChemExpress – indacaterol; TCI America – rifampicin; AA Blocks – gemifloxacin and delafloxacin; Activate Scientific – norfloxacin and levofloxacin; LGC Standards – rifampicin-d_8_, rifampicin N-4′-oxide; – verapamil.

### Construction of *K. pneumoniae* Strain 74189

To eliminate the Inc.F hybrid plasmid pKPN-K7 from strain 70163, a targeted plasmid curing approach was employed using the conjugative CRISPR-Cas9 vector pLCasCureT, as described by Yen and coworkers.^36^ A previously constructed version of this vector, encoding a guide RNA specific to the Inc.F replicon, was conjugated into the parental strain via mating. Following conjugation, expression of Cas9 was induced by the addition of arabinose to activate plasmid targeting. Transconjugants were then selected and screened for plasmid loss. Successful curing of pKPN-K7 was confirmed by the absence of Inc.F replicon-specific PCR amplicons.

### Construction of *K. pneumoniae* Strain 75762

A 20 nucleotide guide RNA (N20) adjacent to a PAM site within the *qnrB1* locus was designed and cloned into the pSGKP plasmid, following the method of Wang and colleagues.^77^ The parental *K. pneumoniae* strain 70163 was cotransformed with three components: the pCasKP plasmid encoding the Cas9 nuclease and the lambda red recombination system, the pSGKP plasmid carrying the guide RNA, and a separate double-stranded DNA repair template containing homologous flanking sequences. This approach enabled the precise deletion of *qnrB1*. Gene deletion was confirmed by Sanger sequencing.

### Alamar Blue Assay to Determine MIC of Drugs Against Bacterial Strains

A broth microdilution assay was performed to determine the MIC of drugs tested against strains of *E. coli*, *A. baumannii*, *K. pneumoniae*, and *P. aeruginosa*. Assays were performed in two biological replicates and the MIC of each drug was determined in duplicate wells. Control wells contained DMSO. An overnight culture of bacteria was subcultured 1:100 in fresh LB media and grown at 37 °C and 180 rpm until reaching an OD_600_ of 0.2 – 0.4. The bacterial culture was then diluted 1:1000 in LB media and added to a 96-well microtiter plate containing different concentrations of serially diluted drug (final volume 100 μL). The plate was grown at 37 °C without shaking. After 24 h, the MIC was determined using either visual inspection of the cell pellet or via the addition of Alamar blue (Invitrogen). In the case of the later, 10% Alamar blue was added to the cultures and returned to the incubator for 3 h. The MIC was determined as the lowest concentration of drug resulting in complete growth inhibition by the chosen method of observation.

### Intrabacterial Drug Accumulation and Metabolism Assay (IBDM)

The IBDM assay was performed with the appropriate Gram-negative bacterial strain with select drugs. All solutions added to cell pellets were prechilled over ice and all centrifugations were performed at 4 °C to minimize further metabolic processes postsample collection. An overnight culture of bacteria was subcultured 1:100 in fresh LB media and grown at 37 °C and 180 rpm until mid log phase (OD_600_ 0.5 – 0.6). A DMSO treated culture was used as the control.

Single-compound studies were typically performed at 20 and/or 10 μM compound; these values are significantly above the LLOD of all tested compounds to ensure reliable quantification. The exceptions may be found for Figure 4 (rifampicin at 1.0 and 10 μM) and Figure 5 (rifampicin at 1.0 μM) given that rifampicin intrabacterial levels were quantifiable at this lower concentration. At the appropriate time point (e.g., 10, 30, and/or 60 min), the OD_600_ of the culture was measured and at least three 1 mL samples were collected and centrifuged at 7,500 rpm for 5 min. The cells were then washed with ice cold 0.85% NaCl solution. The resulting cell pellet was quenched with an ice-cold mixture of 2:2:1 CH_3_OH:CH_3_CN:H_2_O (MAW). The resulting solution was then lysed by four cycles of freeze–thaw (5 min freeze/30 s thaw) in a dry ice/acetone bath. The cell lysate was subsequently centrifuged at 13,000 rpm for 5 min. 700 μL of supernatant were then transferred to a 0.22-μm filter tube and centrifuged at 13,000 rpm for 10 min to filter cellular debris. Samples were stored in a −80 °C freezer until LC/MS analysis.

### LC/MS Analysis of Biological Lysates

Accumulation was measured using an Agilent Infinity II liquid chromatography system coupled with an Agilent 6125 single quadrupole mass spectrometer. Liquid chromatography separation was achieved using an Agilent Poroshell 120 C-18 column with 1.9 μm particle size, 2.1 mm internal diameter, and 50 mm length. The solvent system consisted of CH_3_CN and H_2_O supplemented with 0.1% formic acid. The gradient involved four main phases: 1) an isocratic phase from 0 – 0.3 min at 5% CH_3_CN, followed by 2) 0.3 – 0.6 min increasing CH_3_CN to 30%, 3) 0.6 – 3.3 min increasing CH_3_CN from 30% to 95%, and 4) an isocratic phase from 3.3 – 3.6 min at 95% CH_3_CN. This was followed by a postrun for 1 min at 95% H_2_O. The flow rate was set to 0.5 mL/min, and the column temperature was set to 40 °C. These parameters were held constant for all drugs tested. For mass spectrometry, all analyses were performed using an API-ES ion source in positive polarity. The following spray chamber parameters were used: nebulizer pressure of 40 psi, capillary voltage of 3000 V, drying gas temperature of 350 °C, and drying gas flow of 12.0 L/min. Two main methods were used to quantify intrabacterial accumulation. First, a scan method ranging from *m*/*z* 100 – 1000 was used to investigate the retention time of the drug being tested and its ionization. Then a selective ion monitoring (SIM) method was employed to maximize sensitivity. Each SIM method was set up to search for two target masses: the drug tested and the internal standard. For example, the SIM method for rifampicin searched for *m*/*z* 823 for rifampicin and *m*/*z* 831 for rifampicin-d_8_. For all other drugs, verapamil was used as the internal standard (*m*/*z* 455).

### Calculation of Drug Accumulation from Ion Counts

To quantify drug accumulation expressed as nmols/OD_600_, drug calibration curve samples were prepared and submitted for LC/MS analysis on the same day the biological samples were run on the instrument to control for day-to-day variations in instrument sensitivity. Ten – 15 serial dilutions (typically, 10 – 0.00061 μM) of drug were prepared in 250 μL of 2:2:1 CH_3_CN/MeOH/H_2_O or in bacterial cell lysate. 5.0 μL from a stock solution of internal standard (typically ranging from 10 – 100 μM) was added to all calibration curve samples. The lower limit of detection (LLOD) for each compound was defined as the lowest concentration with a signal-to-noise ratio greater than 3 (Table S6). Peak areas obtained from LC/MS analysis were then plotted using Microsoft Excel 365 and the slope of the best fitting regression line (m) was used in eq 1 to calculate nmols of drug from peak area obtained from biological samples.

1 $$\frac{x}{y}\times \frac{1}{m}\times 1000\times 0.001\times \frac{1}{OD_{600}}=X\frac{\mathrm{nmol}}{OD_{600}}$$

eq 1 calculates X as the drug accumulation expressed in nmol/OD_600_, where “x” and “y” represent the peak areas of drug and internal standard, respectively. “m” represents the slope of the regression line obtained from the calibration curve. The slope was calculated from the calibration curve samples in biological lysates over MAW whenever possible. Since the ion count generated by the LC/MS is a unitless measure, x/y ratio was divided by “m” to afford a number with μM as its units, which was then multiplied by 1000 to convert it to nM. Then, the number was multiplied by 0.001 (to account for the 1 mL sample volume) to yield a final number with nmol units. The final step was to normalize nmols by OD_600_ of the bacterial culture at each time point to yield a final number with units nmol/OD_600_. Comparison of the calibration curve slope determined for each drug in either 2:2:1 CH_3_CN/MeOH/H_2_O or the lysate from *E. coli* MG1655 grown to OD_600_ ∼ 0.5 – 0.6 showed a negligible to modest effect^78^ of the biological matrix on the calculated slope (Table S7). Thus, when conducting single-compound and high-throughput IBDM experiments with Gram-negative bacteria other than *E. coli*, we determined the calibration curve slope using a 2:2:1 CH_3_CN/MeOH/H_2_O solution of the drug.

### Identification of Rifampicin Metabolites Using an LC-TOF System

To search for metabolites of rifampicin, an Agilent 1260 Infinity II liquid chromatography, fitted with an InfinityLab Poroshell 120 EC-C18 column (2.1 × 100 mm, 2.7 μm) and coupled with an Agilent 6230 time-of-flight (TOF) mass spectrometer, was used to analyze the IBDM samples. LC and spray chamber parameters were identical to the LC/MS conditions listed above with the exception of a longer run time (22 min) to accommodate for the lower flow rate (0.3 mL/min) used in the 1260 Infinity II LC system. The Agilent Technologies MassHunter Quant software version 10.0 and the Agilent Personal Compound Database and Library (PCDL version 8.0) were used to search for the putative metabolite (Table S1) in the rifampicin treated sample (200 μM) and the DMSO treated control. Candidate metabolites were first identified as transformations of the parent compound that were observed in drug-treated samples and not in the DMSO-treated control. Second, they were confirmed by matching their *m*/*z* and retention time to the commercially obtained standard.

### Checkerboard Assay for Assessing Synergy of Rifampicin with Indacaterol

A checkerboard assay, as previously described,^79^ assessed interactions between rifampicin and indacaterol with the *E. coli* MG1655 strain. An overnight culture of bacteria was subcultured 1:100 in LB media and incubated at 37 °C and 180 rpm until an OD_600_ of 0.5 was reached. The bacterial culture was then diluted 1:1000 in LB media and added to the wells in a 96-well microtiter plate containing indacaterol (0 – 800 μM) and rifampicin (0 – 50 μM) concentrations in serial dilutions. The plate was then incubated without agitation at 37 °C for 24 h. 10% Alamar blue (Invitrogen) was then added to the plate which was returned to the incubator for 3 h. The MIC was determined as the lowest concentration of drug resulting in complete growth inhibition by visual inspection. Drug interactions were determined by calculating fractional inhibitor index (FICI) using eq 2, where “A” represents MIC of drug A in combination and “B” represents MIC of drug B in combination (FIC ≤ 0.5, synergy; 0.5 < FIC < 4.0, neither synergy nor antagonism; FIC ≥ 4.0, antagonism).^31^

2 $$\mathrm{FIC}\mathrm{Index}=\frac{A}{\mathrm{MI}C_{A}}+\frac{B}{\mathrm{MI}C_{B}}$$

### Cell Staining

Changes in *E. coli* outer membrane permeability as modulated by indacaterol and/or rifampicin were quantified by flow cytometry using TO-PRO-3 (Invitrogen). A culture of *E. coli* MG1655 was diluted 1:100 in LB media and grown at 37 °C with shaking (180 rpm) until an OD_600_ of 0.50 – 0.60 was reached. To the cells was then added DMSO (1% in LB media) as a negative control, 2.5 μM polymyxin B as a positive control, or the tested concentrations of rifampicin and/or indacaterol. To maintain consistency with our IBDM studies, we incubated the bacterial cells with drugs or control for 1 h. Cells were then pelleted at 3,000 rpm for 10 min at 4 °C and then washed with PBS twice. The supernatant was discarded, and the cells were diluted in fresh PBS to afford an OD_600_ = 0.01 (ca. 10^6^ CFU/mL).^80^ TO-PRO-3 (1.0 μM) was added to the cells, and the cells were incubated with the dye at rt in the dark for 10 min.

### Flow Cytometry

Flow cytometry assays were conducted with the BD LSRII (BD Biosciences). TO-PRO-3 was detected using APC with an excitation of 633 nm and a 660/20 bandpass filter. Results were analyzed using BD FlowJo software (version 10.7.2, BD Biosciences).

### High-Throughput Intrabacterial Drug Accumulation and Metabolism (htIBDM) Assay

Initially Z’^32^ was determined as follows. An *E. coli* MG1655 culture was grown in LB media at 37 °C with shaking at 180 rpm to an OD_600_ of 0.55 – 0.65. 1.1 mL of the culture was then transferred to each well of a 96-well deep well plate (Agilent # 201240–100) and sealed with a seal mat (Agilent #201158–100). Ten μM of doxycycline was added to all odd-numbered wells, and DMSO was added to all even-numbered wells. The plate was then incubated at 37 °C with shaking at 280 rpm for 1 h. 100 μL from each well in the original plate was transferred to a separate 96-well plate to obtain OD_600_ measurements using an Agilent BioTek Synergy HTX Multimode Reader. The original plate was then centrifuged at 4,000 rpm and 4 °C for 10 min, washed twice with ice-cold 0.85% aqueous NaCl, and quenched with ice-cold 2:2:1 CH_3_CN/CH_3_OH/H_2_O. Bacterial cells were then lysed by one cycle of freeze–thaw and 3 × 5 min cycles of sonication followed by centrifugation for 10 min at 4,000 rpm. The cell lysate (250 μL) was subsequently filtered using a filter plate (Agilent #203980–100), and flow-through was collected in a collection plate (Nunc #260251). The resulting filtrate was used for LC/MS analysis as described above. Subsequent htIBDM studies were typically performed at 20 μM compound, although concentrations of 10 μM (Figure S11) and 40 μM (Figure 8) were also utilized.

### Statistical Analyses

All IBDM assays were performed in at least triplicate with two biological replicates. All statistical analysis was performed using GraphPad Prism (version 10.0.0) and are detailed in the caption for each figure. The post hoc test used for each statistical analysis was selected based on the type of comparison performed and as recommended by GraphPad Prism.

## Supplementary Material





## Data Availability

The data sets generated during and/or analyzed during the current study are available in the Supporting Information.

## Notes

Chemical formulas and exact masses for rifampicin and its investigated metabolite, MICs of rifampicin and rifampicin N-4′-oxide against *E. coli* MG1655, MIC values of rifampicin against select Gram-negative ESKAPE strains, Molecular weight (MW) and h_logP values for compounds tested, Genotypes of clinical isolate 70163 and its plasmid-cured 74189 and *qnrB1* knockout 75762 derivative strains, Lower limit of detection (LLOD) for drugs used in this study, Effect of matrix on the calibration curves for moxifloxacin, doxycycline, and rifampicin (Tables S1 – S7) and Accumulation of rifampicin in *E. coli* MG1655 in three different culture volumes, Accumulation of rifampicin and doxycycline in *E. coli*MG1655 at three different time points, Doxycycline and rifampicin accumulation in *E. coli* MG1655 normalized by OD_600_ versus colony-forming units (CFUs), Moxifloxacin, doxycycline, and rifampicin time-dependent accumulation in *E. coli* MG1655, Moxifloxacin, doxycycline, and rifampicin dose-dependent accumulation in *E. coli* MG1655 at t = 60 min, Drug minimum inhibitory concentration (MIC) and intrabacterial accumulation fold-changes in the MG1655 and *ΔtolC* strains, chromatograms of rifampicin and its N-4′-oxide metabolite in cell lysate compared to a commercial standard, checkerboard assay showing interaction between rifampicin and indacaterol with respect to the *E. coli* MG1655 strain, DMSO treated *E. coli* MG1655 cells in the presence of TO-PRO-3 dye, Single compound IBDM of rifampicin, moxifloxacin, novobiocin, ciprofloxacin and doxycycline in *E. coli* MG1655, effect of number of washes on the accumulation of novobiocin in the htIBDM assay with *E. coli* MG1655, predicted physicochemical properties of six fluoroquinolones compared to their accumulation. (Figures S1 – S12). Supplementary_File_1 (PDF)
Overview of IBDM calculations (XLSX)
