# Supplementary material for: The Quantification of Drug Accumulation within Gram-Negative Bacteria
Source: ACS Infect Dis. 2025 Dec 24;12(1):410–24. doi: 10.1021/acsinfecdis.5c00921 (PMC12797233; doi:10.1021/acsinfecdis.5c00921)
Supplement: Supplementary file 1 [file id5c00921_si_001.pdf]

Supplementary Information

The Quantification of Drug Accumulation within Gram-Negative Bacteria

Amir George,[1,†] Shivangi,[1,†] Alexandra Bozan,[1] Kendra Spencer,[1] Austin J. Terlecky,[2] Yong-Mo Ahn,[1] Pamela R. Barnett,[1] Barry N. Kreiswirth,[2] Joel S. Freundlich[1,3,*]

[1]Department of Pharmacology, Physiology, and Neuroscience, Rutgers University – New Jersey Medical School, Newark, New Jersey, USA.

[2]Hackensack Meridian Health Center for Discovery & Innovation, Nutley, New Jersey, USA.

[3]Division of Infectious Disease, Department of Medicine and the Ruy V. Lourenco Center for the Study of Emerging and Re-emerging Pathogens, Rutgers University – New Jersey Medical School, Newark, New Jersey, USA.

† Contributed equally

* Address correspondence to Joel S. Freundlich (freundjs@rutgers.edu)

Table of Contents

Figure S1. Accumulation of rifampicin in *E. coli* MG1655 in three different culture volumes. Page S4

Figure S2. Accumulation of rifampicin and doxycycline in *E. coli* MG1655 at three different time points. Page S5

Figure S3. Doxycycline and rifampicin accumulation in *E. coli* MG1655 normalized by OD_{600} versus colony-forming units (CFUs). Page S6

Figure S4. Moxifloxacin, doxycycline, and rifampicin time-dependent accumulation in *E. coli* MG1655. Page S7

Figure S5. Moxifloxacin, doxycycline, and rifampicin dose-dependent accumulation in *E. coli* MG1655 at t = 60 min. Page S8

Figure S6. Drug minimum inhibitory concentration (MIC) and intrabacterial accumulation fold-changes in the MG1655 and *ΔtolC* strains. Page S9

Figure S7. Chromatograms of rifampicin and its N-4′-oxide metabolite in cell lysate compared to a commercial standard. Page S10

Figure S8. Checkerboard assay showing interaction between rifampicin and indacaterol with respect to the *E. coli* MG1655 strain. Page S11

Figure S9. DMSO treated *E. coli* MG1655 cells in the presence of TO-PRO-3 dye. Page S12

Figure S10. Single compound IBDM of rifampicin, moxifloxacin, novobiocin, ciprofloxacin and doxycycline in *E. coli* MG1655. Page S13

Figure S11. Effect of number of washes on the accumulation of novobiocin in the htIBDM assay with *E. coli* MG1655. Page S14

Figure S12. Predicted physicochemical properties of six fluoroquinolones compared to their accumulation. Page S15

Table S1. Chemical formulas and exact masses for rifampicin and its investigated metabolite. Page S16

Table S2. MICs of rifampicin and rifampicin N-4′-oxide against *E. coli* MG1655. Page S17

Table S3. MIC values of rifampicin against select Gram-negative ESKAPE strains. Page S18

Table S4. Molecular weight (MW) and h_logP values for compounds tested. Page S19

Table S5. Genotypes of clinical isolate 70163 and its plasmid-cured 74189 and *qnrB1* knockout 75762 derivative strains. Page S20

Table S6. Lower limit of detection (LLOD) for drugs used in this study. Page S21

Table S7. Effect of matrix on the calibration curves for moxifloxacin, doxycycline, and rifampicin. Page S22

It should be noted that all data for the IBDM runs discussed in this publication may be found in the file: Supplementary_File_1.XLSX

Figure S1. Accumulation of rifampicin in *E. coli* MG1655 in three different culture volumes. Data are shown as mean ± SD for a representative of two independent experiments each conducted in triplicate of rifampicin (Rif; 10 µM) incubated with bacteria with three different culture volumes (15, 25, and 125 mL) at t = 60 min. A Kruskal-Wallis test with Dunn post-hoc test was performed, and no statistically significant difference was found between rifampicin accumulation in all three culture volumes. ns $p>0.05$.

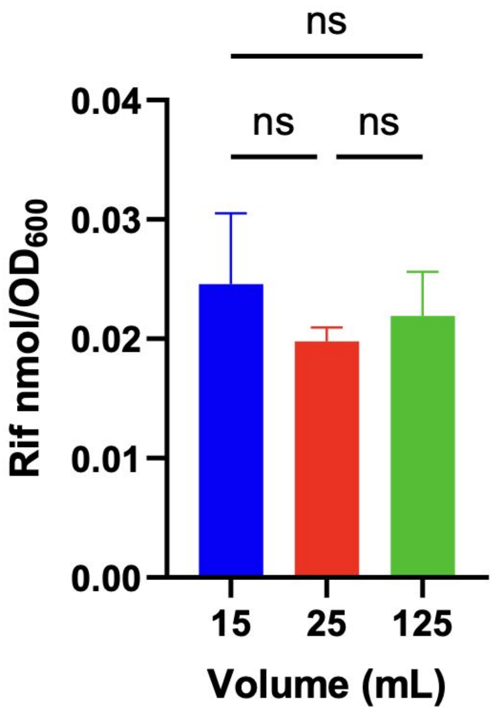

Figure S2. Accumulation of rifampicin and doxycycline in *E. coli* MG1655 at three different time points. Data are shown as mean ± SD for a representative of two independent experiments each conducted in quadruplicate for a) rifampicin (10 μM) and b) doxycycline (10 μM) incubated with a 25 mL culture of *E. coli* MG1655 at 3 different time points (10, 30, and 60 min). An ordinary one-way ANOVA with Tukey post-hoc test was performed, and no statistically significant difference was found for the accumulation for each drug with respect to the time points. ns p>0.05.

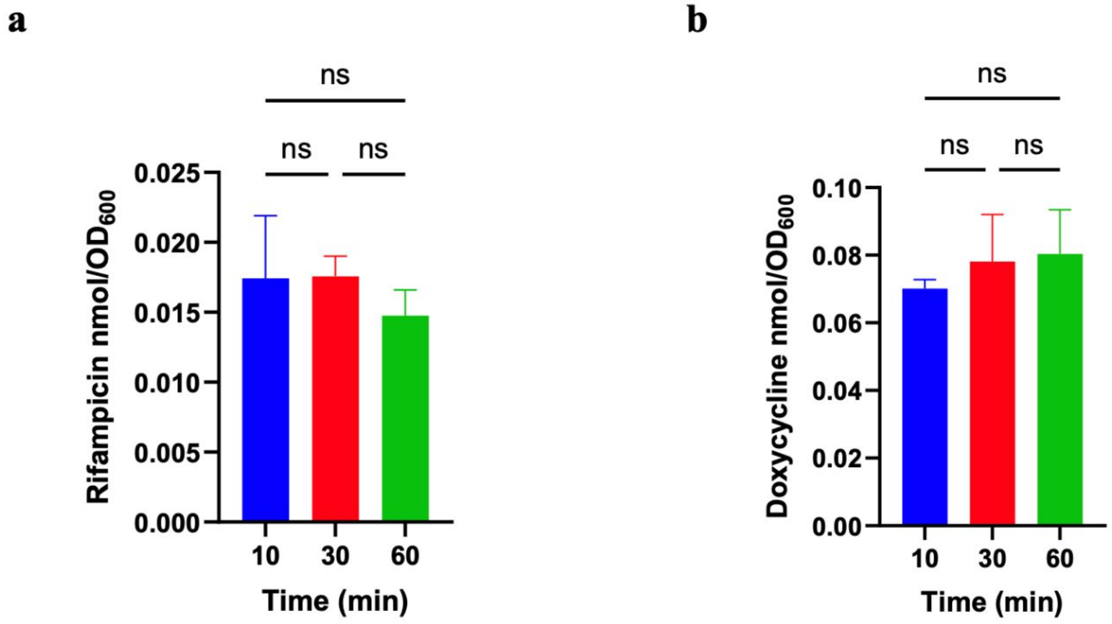

Figure S3. Doxycycline and rifampicin accumulation in *E. coli* MG1655 normalized by OD_{600} versus colony-forming units (CFUs). Doxycycline and rifampicin accumulation at 10 µM compound and t = 60 min, normalized by a) $\log_{10}$ CFUs compared to b) OD_{600}. Data are shown as mean ± SD representative of two independent experiments each conducted in quadruplicate. p-values were determined by two-way ANOVA with Šídák post hoc test. ns p>0.05, * p<0.05, ** p<0.01, *** p<0.001, **** p<0.0001.

a

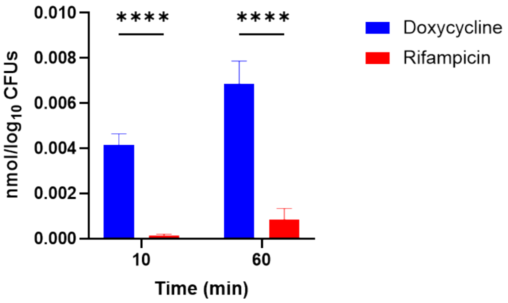

b

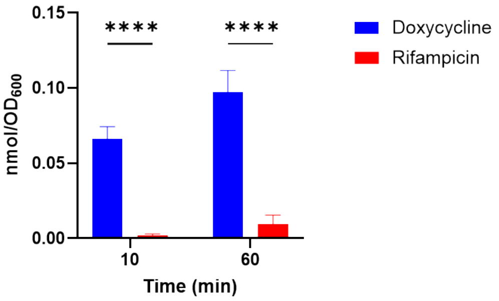

Figure S4. Moxifloxacin, doxycycline, and rifampicin time-dependent accumulation in *E. coli* MG1655. a) Moxifloxacin time-dependent accumulation at 10 and 20 µM. b) Doxycycline time-dependent accumulation at 10 and 20 µM. c) Rifampicin time-dependent accumulation at 10 and 20 µM. Data are shown as mean ± SD for a representative of two independent experiments each conducted in quadruplicate. The amount of accumulated compound as the number of moles was normalized by cell number as approximated by OD_{600}. p-values were determined by two-way ANOVA with Tukey post hoc test. ns p>0.05, * p<0.05, ** p<0.01, *** p<0.001, **** p<0.0001. For each drug at each of the two concentrations assessed, the following pairwise comparisons demonstrated ns p>0.05 : 10 min vs. 30 min, 10 min vs. 60 min, and 30 min vs. 60 min.

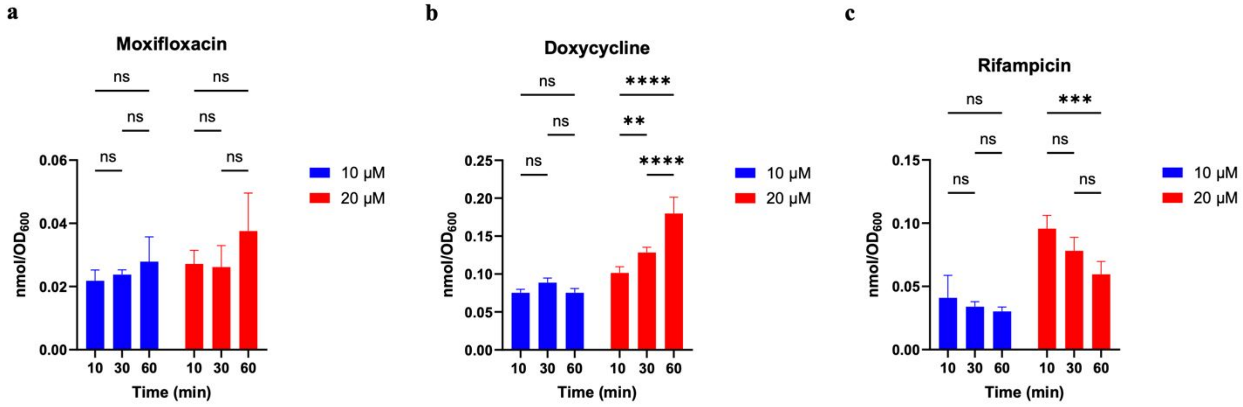

Figure S5. Moxifloxacin, doxycycline, and rifampicin dose-dependent accumulation in *E. coli* MG1655 at t = 60 min. Data are shown as mean ± SD for a representative of two independent experiments each conducted in quadruplicate for a) moxifloxacin, b) doxycycline, and c) rifampicin at 10 and 20 µM. The amount of accumulated compound as the number of moles was normalized by cell number as approximated by OD_{600}. p-values were determined by unpaired t-tests comparing the two concentrations for each drug. ns p>0.05, * p<0.05, ** p<0.01, *** p<0.001, **** p<0.0001.

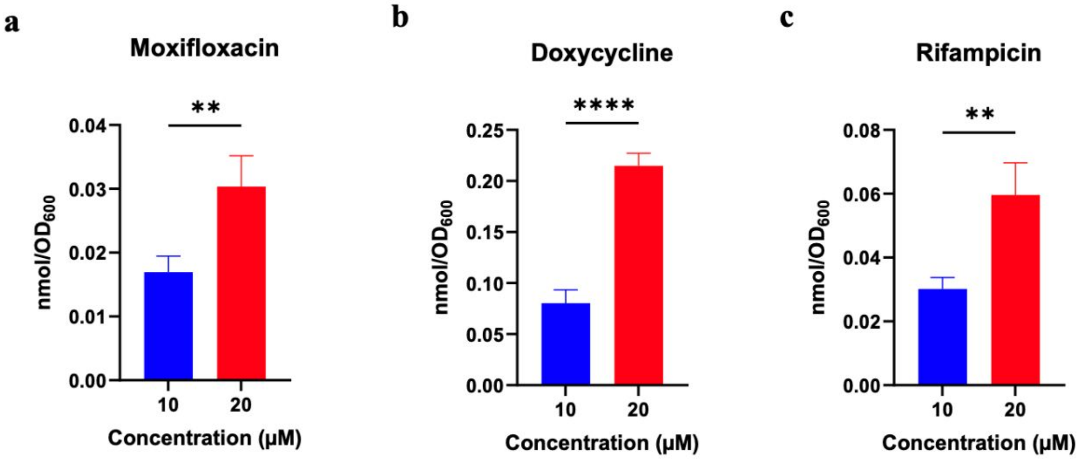

Figure S6. Drug minimum inhibitory concentration (MIC) and intrabacterial accumulation fold-changes in the MG1655 and *ΔtolC* strains. Accumulation values at 10 µM drug and MICs are shown in Figure 3 and Table 1, respectively. Although a monotonic relationship between the two variables is visually suggested by this plot, a correlation statistic for these data was not determined given consideration of the small number of points (n = 3), which does not enable testing of the strength of this relationship.[1-4]

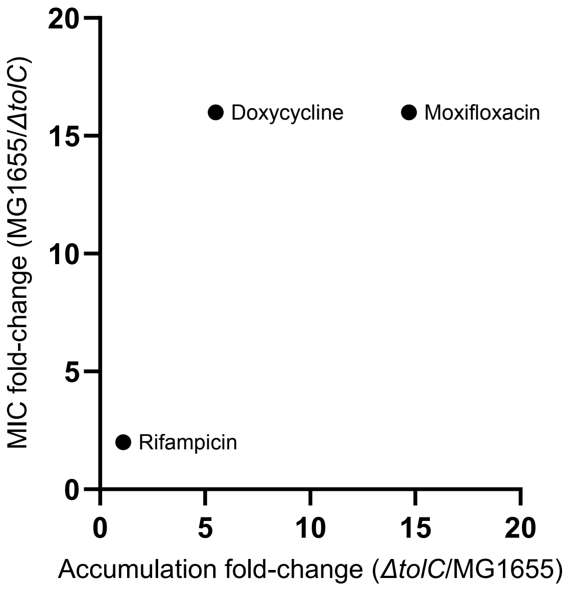

Figure S7. Chromatograms of rifampicin and its N-4′-oxide in cell lysate compared to a commercial standard. Chromatograms of rifampicin N-4′-oxide authentic standard (red), rifampicin N-4′-oxide detected in *E. coli* MG1655 (green) and the co-injection of the biological sample and rifampicin N-4′-oxide authentic standard (orange).

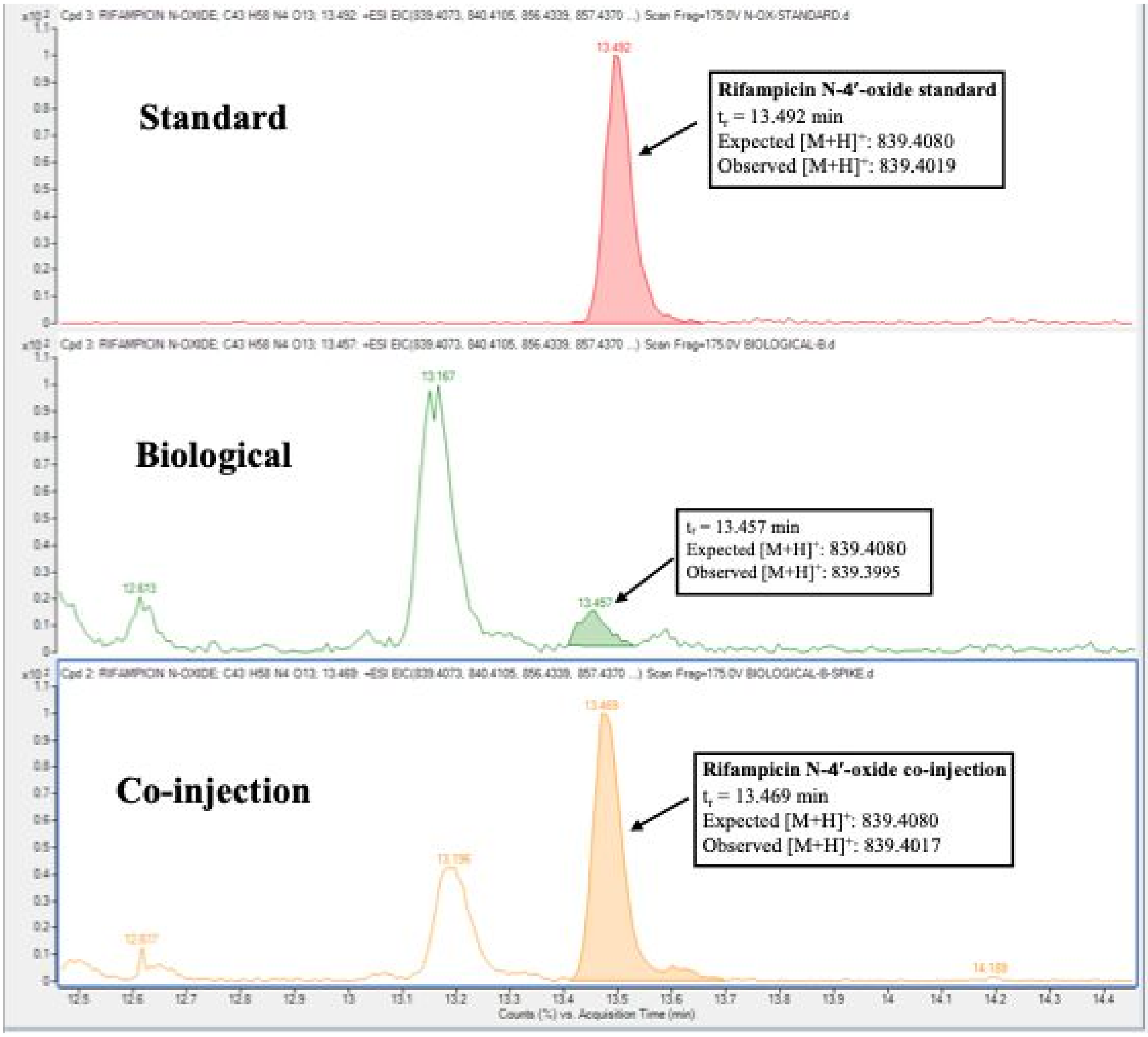

Figure S8. Checkerboard assay showing interaction between rifampicin and indacaterol with respect to the *E. coli* MG1655 strain. Alamar blue was used to determine the MIC of each drug as the lowest concentration with no cell growth by visual inspection. Drug interactions were determined by calculation of the fractional inhibitor (FIC) index (FIC ≤ 0.5, synergy; 0.5 < FIC < 4.0, neither synergy nor antagonism; FIC ≥ 4.0, antagonism). The rifampicin MIC in combination was ≤0.78 μM, the indacaterol MIC in combination was 50 μM, and, thus, the FIC index was calculated to be ≤0.19.

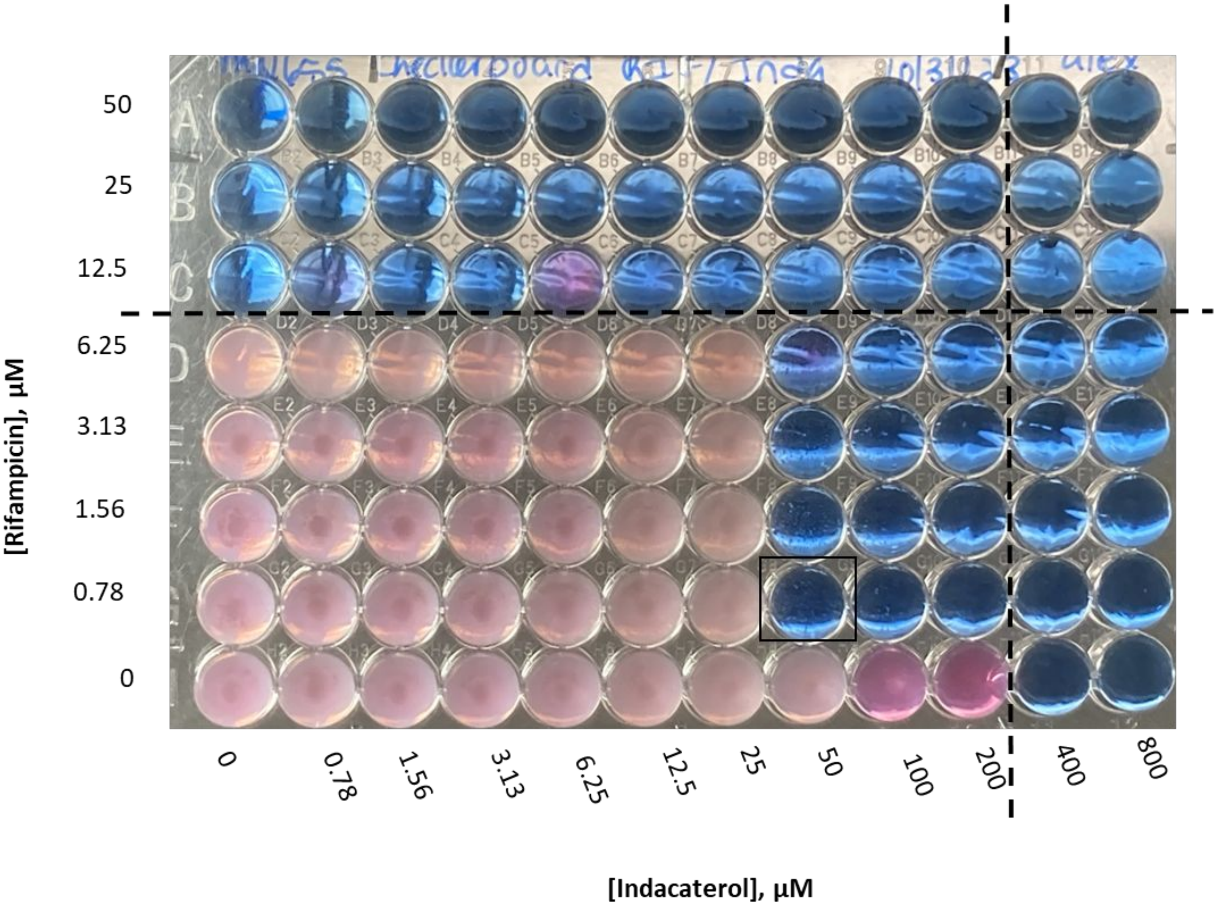

Figure S9. DMSO treated *E. coli* MG1655 cells in the presence of TO-PRO-3 dye. These data represent the negative control for dye uptake where the percent of cells that took up the dye is shown. The y-axis represents the side scatter area, and the x-axis represents the fluorescence of TO-PRO-3. Results are representative of three independent experiments.

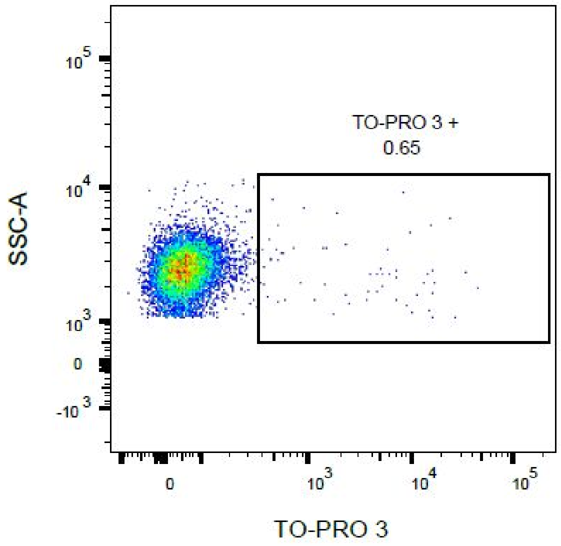

Figure S10. Single-compound IBDM assay accumulation of rifampicin, moxifloxacin, novobiocin, ciprofloxacin and doxycycline in *E. coli* MG1655. Data are shown as mean ± SD representative of two independent experiments each conducted in quadruplicate for the drug concentration of 20 µM and at t = 60 min. The amount of accumulated compound as the number of moles was normalized by cell number as approximated by OD_{600}. p-values were determined by a one-way ANOVA with Tukey post hoc test. ns p>0.05, * p<0.05, ** p<0.01, *** p<0.001, **** p<0.0001.

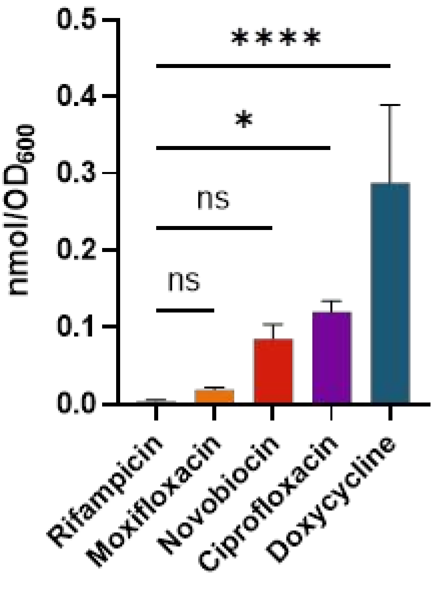

Figure S11. Effect of number of washes on the accumulation of novobiocin in the htIBDM assay with *E. coli* MG1655. Bacteria were incubated with 10 μM novobiocin and a time point of 60 min was used. Data are shown as mean ± SD conducted in quadruplicate. The amount of accumulated compound as the number of moles was normalized by cell number as approximated by OD_{600}. p-values were determined by a Kruskal-Wallis test with Dunn post hoc test. ns $p>0.05$, * $p<0.05$, ** $p<0.01$.

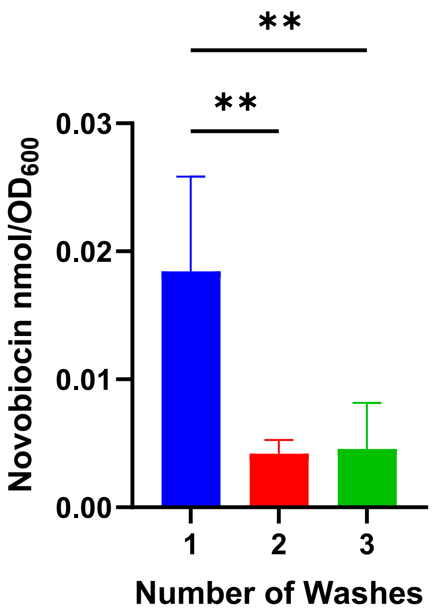

Figure S12. Predicted physicochemical properties of six fluoroquinolones compared to their accumulation. The selected properties were chosen based on previous reports correlating hydrophobicity and molecular weight to accumulation in Gram-negative bacteria and the eNTRy rules developed by Richter et al. Accumulation values (nmol/OD_{600}) of six fluoroquinolones in *K. pneumoniae* strain 70163 were plotted against a) molecular weight (MW), b) predicted octanol-water partition coefficient (h_logP), c) number of rotatable bonds, d) globularity and e) amphiphilic moment (vsurf_A). Physicochemical properties were computed in the MOE software package (Version 2024.06).

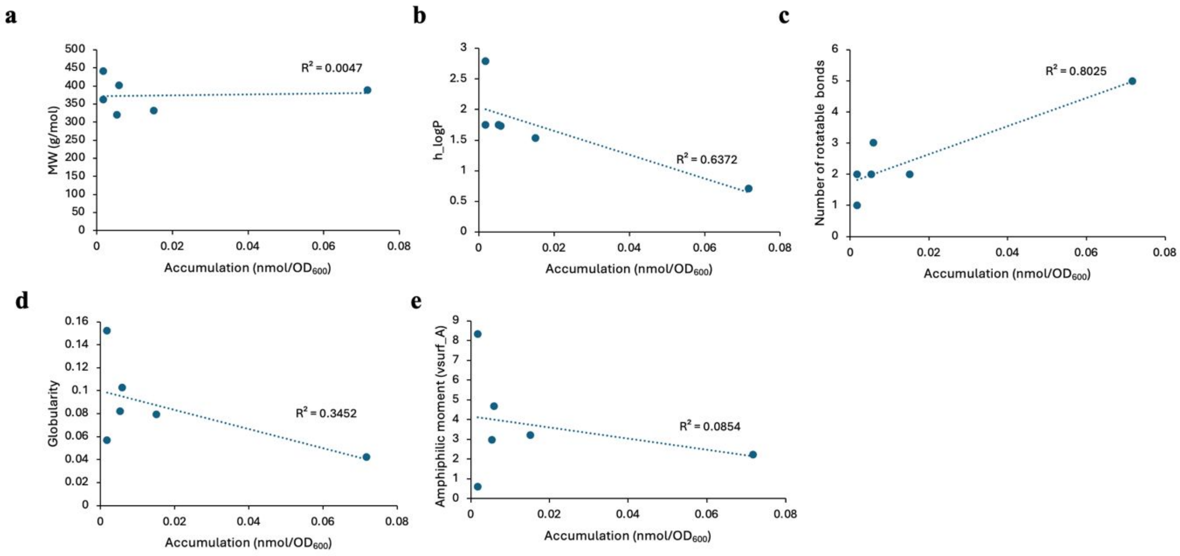

Table S1. Chemical formulas and exact masses for rifampicin and its investigated metabolite.

Molecule	Chemical Formula	Exact Mass (amu)	Expected $[M+H]^+$
rifampicin	$C_{43}H_{58}N_4O_{12}$	822.4051	823.4131
rifampicin N-4′-oxide	$C_{43}H_{58}N_4O_{13}$	838.4000	839.4080

Table S2. MICs of rifampicin and rifampicin N-4′-oxide against *E. coli* MG1655. Each MIC value represents the average value from a minimum of two independent experiments.

**Compound**	**MIC against *E. coli* MG1655 (µM)**
rifampicin	12
rifampicin N-4′-oxide	>200

Table S3. MIC values of rifampicin against select Gram-negative ESKAPE strains. MIC values were calculated as the mean of two independent experiments, each performed in duplicate.

**Bacterial Strain (ATCC#)**	**Rifampicin MIC (µM)**
P. aeruginosa (HER-1018)	12
K. pneumoniae (BAA 2146)	50
A. baumannii (19606)	0.78

Table S4. Molecular weight (MW) and h_logP values for compounds tested. Descriptors were generated with the MOE software package (Version 2024.06).

Drug	MW (g/mol)	h_logP
rifampicin	822.9	3.6
novobiocin	612.6	3.4
doxycycline	444.4	0.47
ciprofloxacin	331.3	1.5
moxifloxacin	401.4	1.7

Table S5. Genotypes of clinical isolate 70163 and its plasmid-cured 74189 and *qnrB1* knockout 75762 derivative strains. Curing of the IncF hybrid plasmid (pKPN-K7-1) facilitated removal of all resistance genes harbored on it. 70163 contains chromosomal mutations conferring fluoroquinolone resistance that persist within the plasmid-cured strain 74189. 75762 is the *qnrB1* knockout of the 70163 strain.

Strain	Sequence Type (ST)	Plasmid Content	Plasmid Encoded Aminoglycoside Resistance	Plasmid Encoded Fluoroquinolone Resistance	Chromosomal Resistance Mutations
70163	ST307	IncF: pKPN-K7-1	*aac(6')-Ib-cr*	*qnrB1*	GyrA-83I; ParC-80I
74189	ST307	Plasmid-free			GyrA-83I; ParC-80I
75762	ST307	IncF: pKPN-K7-1	*aac(6')-Ib-cr*		GyrA-83I; ParC-80I

Table S6. Lower limit of detection (LLOD) for drugs used in this study. Calibration curves were prepared using twofold serial dilutions in 2:2:1 MeOH:ACN:H_2O. For compounds used in the single-compound format IBDM studies, dilutions ranged from 10 to 0.000305 µM, while for compounds used in the htIBDM studies, dilutions ranged from 10 to 0.00488 µM. Samples were analyzed by LC/MS, and the lower limit of detection (LLOD) for each compound was defined as the lowest concentration with a signal-to-noise ratio greater than 3.

Drug	LLOD (µM)
doxycycline	0.00183
rifampicin	0.00366
novobiocin	0.00183
ciprofloxacin	0.00305
moxifloxacin	0.00183
norfloxacin	0.00488
delafloxacin	0.00488
gemifloxacin	0.00488
levofloxacin	0.00488
ofloxacin	0.00488

Table S7. Effect of matrix on the calibration curves for moxifloxacin, doxycycline, and rifampicin. Calibration curves were constructed in $CH_3OH:CH_3CN:H_2O$ (MAW; 2:2:1) and in *E. coli* MG1655 biological lysate in MAW. The effect of the matrix was determined by dividing the slope of the calibration curve (sensitivity) in biological lysate by the slope of the calibration curve in MAW. Values are expressed as the mean from three experiments.

**Drug**	**Sensitivity in MAW (S_M)**	**Sensitivity in Biological Lysate (S_B)**	**S_B / S_M**
moxifloxacin	0.67	0.55	0.82
doxycycline	0.35	0.36	1.0
rifampicin	0.34	0.43	1.3

REFERENCES

(1) Bujang, M. A. An elaboration on sample size determination for correlations based on effect sizes and confidence interval width: a guide for researchers. *Restorative Dentistry & Endodontics* **2024**, *49* (2).

(2) Cao, Y.; Chen, R. C.; Katz, A. J. Why is a small sample size not enough? *The Oncologist* **2024**, *29* (9), 761–763.

(3) Makin, T. R.; Orban de Xivry, J.-J. Ten common statistical mistakes to watch out for when writing or reviewing a manuscript. *Elife* **2019**, *8*, e48175.

(4) Bonett, D. G.; Wright, T. A. Sample size requirements for estimating Pearson, Kendall and Spearman correlations. *Psychometrika* **2000**, *65* (1), 23–28.
